# The Influence of Aphasia Type and Severity on Sentence Comprehension after Left Hemisphere Stroke

**DOI:** 10.1111/1460-6984.70245

**Published:** 2026-04-09

**Authors:** Courtney Gilman, Arianna N. LaCroix

**Affiliations:** ^1^ Department of Speech, Language, and Hearing Sciences Purdue University West Lafayette Indiana USA

**Keywords:** aphasia, communication disorder, language assessments, language comprehension, stroke rehabilitation, syntax

## Abstract

**Background:**

Sentence comprehension relies on the integrity of a left‐lateralized language network that is frequently disrupted following left hemisphere stroke. While comprehension deficits are well documented in Broca's and Wernicke's aphasia, less is known about how sentence comprehension varies across the broader aphasia spectrum, including types traditionally viewed as comprehension‐intact, such as anomic and latent aphasia.

**Aims:**

This study examined how sentence comprehension varies as a function of aphasia type and severity, with particular focus on comprehension of syntactically complex, non‐canonical sentences. We also aimed to characterize comprehension in latent aphasia, an understudied group of stroke survivors who perform within normal limits on standardized assessments but report residual language difficulties.

**Methods and Procedures:**

Eighty‐five adults participated, including 64 individuals with left hemisphere stroke and 21 neurotypical controls. Sentence comprehension was assessed using a sentence–picture matching task containing canonical (subject‐relative) and non‐canonical (object‐relative) sentences. Aphasia type (latent, anomic, Broca's, conduction) and severity (latent, mild, moderate, severe) were determined using the *Western Aphasia Battery–Revised (WAB–R)*. Separate mixed‐effects models were used to examine the effects of aphasia type, severity, and syntactic structure on comprehension accuracy and reaction time.

**Outcomes and Results:**

Sentence comprehension accuracy declined with increasing aphasia severity. Agrammatic comprehension patterns—characterized by poorer performance on non‐canonical than canonical sentences—were observed across all aphasia types, though the degree of impairment varied. The latent aphasia group performed better than the anomic group but worse than controls. The Broca's and conduction aphasia groups showed greater impairments than the anomic group but did not differ from each other.

**Conclusions and Implications:**

Sentence comprehension deficits occur across a wide range of aphasia severities, including in individuals with latent aphasia who score above clinical diagnostic cutoffs. These results underscore the need to assess sentence comprehension across the full aphasia spectrum using syntactically complex sentences, which may help identify subtle impairments that impact functional communication.

**WHAT THIS PAPER ADDS:**

*What is already known on this subject*

- Sentence comprehension deficits in aphasia vary by severity and type, but prior studies have typically examined these factors separately and have rarely included individuals with latent aphasia who fall above diagnostic thresholds. As a result, it remains unclear how sentence comprehension varies across the full spectrum of aphasia.

*What this study adds to existing knowledge*

- This study systematically examined how sentence comprehension accuracy and response times vary with both aphasia type and severity, across canonical and non‐canonical syntactic structures. Findings demonstrate that comprehension deficits are more widespread than previously recognized, extending even to individuals with latent aphasia.

*What are the potential or actual clinical implications of this work?*

- The results underscore the need for assessment tools that incorporate syntactically complex sentences to detect subtle comprehension deficits that may not be captured by standard tests. Tailoring intervention to an individual's syntactic comprehension profile may enhance treatment outcomes.

## 1 Introduction

Sentence comprehension depends on the coordinated activity of a left‐lateralized fronto‐temporo‐parietal network (Friederici 2011, 2012; Hickok and Poeppel 2007). Damage to these regions, such as from left hemisphere stroke, often results in aphasia—a language disorder that disrupts speaking, understanding, reading, and writing to varying degrees. Aphasia severity is one of the strongest predictors of post‐stroke language outcomes, with greater severity associated with more extensive impairments and poorer treatment responses (Fridriksson and Hillis 2021; Kristinsson et al. 2023; Nakagawa et al. 2019; Plowman et al. 2012). This relationship extends to sentence comprehension: individuals with mild aphasia typically outperform those with moderate aphasia, who in turn outperform those with severe aphasia (Klor and Mlchoh 1984; Murray 2018; Sung et al. 2024).

While severity captures the overall extent of language impairment, aphasia type influences the nature and pattern of sentence comprehension deficits. For example, individuals with Broca's aphasia often struggle with non‐canonical structures, sentences that deviate from the canonical subject–verb–object order; a pattern known as agrammatic comprehension (Caplan et al. 2013; Grodzinsky 2000; Love et al. 2008). In Wernicke's aphasia, comprehension difficulties tend to be more pervasive, affecting both canonical and non‐canonical sentences due to disruptions in semantic and lexical processing (Caramazza and Zurif 1976; Clark 2011; Dick et al. 2001; Heilman and Scholes 1976; Shapiro and Levine 1990; Shewan and Canter 1971). In contrast, sentence comprehension in conduction and anomic aphasia is often described as being relatively preserved (Clark 2011; Papathanasiou & Coppens, 2016), although sentences that place heavy demands on working memory or syntactic integration can lead to significant difficulty (Baldo et al. 2008; Caramazza et al. 1981; Caramazza and Zurif 1976; Dick et al. 2001; Friedmann and Gvion 2003; Gvion and Friedmann 2012; Heilman and Scholes 1976). Increasing evidence further suggests that individuals with anomic aphasia may exhibit comprehension deficits resembling those found in Broca's or conduction aphasia (Akinina et al. 2021; Berndt et al. 1997; DeDe 2012, 2013; Dick et al. 2001; Peach et al. 1988; Salis et al. 2021; Shewan and Canter 1971).

Few studies have directly compared sentence comprehension across multiple aphasia types. Those that have, consistently report that comprehension is poorest in Wernicke's aphasia, followed by Broca's, and least impaired in anomic aphasia (Dick et al. 2001; Parisi and Pizzamiglio 1970; Shewan and Canter 1971). Together, these findings highlight that both type and severity shape sentence comprehension profiles in aphasia. Yet these factors are rarely examined simultaneously.

A group historically excluded from studies of sentence comprehension is individuals with latent aphasia, or those who have sustained a left hemisphere stroke but score above the clinical cutoff for aphasia on standardized language assessments. Although considered to have ‘intact’ language, individuals with latent aphasia often report language difficulties that interfere with daily communication, work, and social participation (Cavanaugh and Haley 2020). Empirical evidence supports these self‐reports, revealing measurable deficits in discourse production (Bunker et al. 2025; Dalton and Richardson 2015; DeDe and Salis 2020; Fromm et al. 2017; Salis and DeDe 2022; Zhang et al. 2024), sentence recall (Newcombe and Marshall 1967; Salis et al. 2021), following directions (Boller 1968; Boller and Vignolo 1966), and comprehension of sentences with relative and passive clauses (Martzoukou et al. 2025). Collectively, these findings suggest that language processing in latent aphasia is not entirely intact, underscoring the need for continued investigation into this underrecognized clinical profile.

There are two primary competing theoretical accounts of why sentence comprehension impairments arise in post‐stroke aphasia. Representational accounts propose that difficulty with non‐canonical structures arises from disruptions in syntactic knowledge or in the mapping of structural relations among sentence constituents (e.g., Grodzinsky 1995, 2000). Within this framework, related proposals distinguish between lexical mapping deficits, arising from impaired single word comprehension, and syntactic mapping deficits, in which structural dependencies are not successfully computed (Marshall 1995; Rochon et al. 2005). In contrast, resource‐based accounts emphasize limitations in cognitive capacity that become more apparent as linguistic complexity increases (e.g., Caplan et al. 2007, Caplan et al. 2013; Just and Carpenter 1992). Complementary perspectives further highlight the role of probabilistic expectations, such that less frequent structures impose greater processing demands (Levy 2008; MacDonald and Christiansen 2002; Wells et al. 2009).

Against this theoretical backdrop, the current study examined how sentence comprehension impairments vary as a function of aphasia type and severity, with particular attention to changes across increasing levels of syntactic complexity. Understanding these relationships is essential for improving both assessment and intervention. Specifically, we aimed to: (1) integrate research on aphasia type and severity, which have largely been examined in independent samples; (2) extend previous work by incorporating syntactic complexity as a key variable; and (3) broaden understanding of language impairments in latent aphasia by comparing this group to multiple aphasia types beyond anomic aphasia. We hypothesized that sentence comprehension accuracy would decrease, and response times would increase with greater aphasia severity. Additionally, we expected all aphasia types, including latent aphasia, to show reduced comprehension relative to neurotypical controls, with poorer performance for non‐canonical than canonical sentences across all groups.

## 2 Method

### 2.1 Participants

A retrospective analysis combined data from 64 people with left hemisphere stroke (PWS; 27 female) at least 6 months prior to testing from two studies (*n* = 16 and *n* = 48). Participants ranged in age from 31–82 years (*M =* 59.25, *SD =* 11.11), were native speakers of American English, were right‐handed pre‐stroke, had 16.73 (*SD =* 2.70) years of education on average, and no history of major psychiatric disease or neurological disorder other than stroke. Aphasia type and severity were classified using the *WAB‐R* (Kertesz 2007) and are reported in Table 1. Participants with an aphasia quotient less than 93.8 were determined to have aphasia and were assigned an aphasia type and severity using the *WAB‐R* classifications. Those with an aphasia quotient greater than 93.8 were classified as having latent aphasia.

**TABLE 1:** Participant Demographics.

Subject	Gender	Age	Ed.	PTA^*^	TPS (Mo.)	WAB‐R AQ	WAB‐R	Aphasia	Aphasia
			(Yr.)				Aud Comp	severity	type
1020	M	80	13	42.5	246	99.2	60	Latent	Latent
1002	F	49	17	30	134	97.8	60	Latent	Latent
1013	F	60	21	25	82	96.8	60	Latent	Latent
1004	F	60	13	^**^	48	96	60	Latent	Latent
1015	F	43	17	30	27	95.6	58	Latent	Latent
0024	F	55	16	12.5	98	95.5	59	Latent	Latent
1007	M	58	17	27.5	31	95.5	60	Latent	Latent
1039	M	76	17	16.25	96	95.4	60	Latent	Latent
1018	M	66	21	35	45	95.1	59	Latent	Latent
0006	M	44	16	20	63	95	60	Latent	Latent
0017	M	65	20	16.89	107	93.2	60	Mild	Anomic
0001	M	31	14	16.25	52	93	60	Mild	Anomic
0014	F	57	18	18.75	67	93	59	Mild	Anomic
1019	F	56	19	21.25	438	92.8	59	Mild	Anomic
1041	M	67	19	21.25	37	92	60	Mild	Anomic
1009	M	63	19	31.25	146	91.9	60	Mild	Anomic
1014	F	51	19	7.5	281	89.3	59	Mild	Anomic
0027	M	59	12.5	13.75	6	88.6	58	Mild	Anomic
0030	F	72	9	41.25	81	88.1	60	Mild	Anomic
1003	F	38	19	47.5	64	87.6	60	Mild	Anomic
1045	F	49	17.5	30	117	87.1	60	Mild	Anomic
1033	F	50	17	27.5	47	87	60	Mild	Anomic
1010	M	58	21	40	46	85.7	56	Mild	Anomic
1040	F	63	19	25	62	85.6	60	Mild	Anomic
1038	M	55	17	15	68	84.8	58	Mild	Anomic
1035	M	59	17	25	86	84.1	59	Mild	Anomic
0002	F	49	12	35	91	83.8	60	Mild	Anomic
1025	M	61	21	27.5	137	82.7	59	Mild	Anomic
1006	M	63	17	20	72	81.5	60	Mild	Conduction
1030	M	71	19	50	80	80.6	60	Mild	Anomic
1029	F	61	19	23.75	33	79.2	60	Mild	Broca's
1028	M	62	19	18.75	237	78.9	56	Mild	Anomic
1005	F	31	17	37.5	81	77.9	57	Mild	Anomic
1047	M	72	13	17.5	178	77.2	60	Mild	Anomic
1037	M	67	19	23.75	36	76.2	59	Mild	Anomic
1042	M	59	13	17.5	61	74.9	59	Moderate	Broca's
1043	M	54	17	3.75	94	74.6	57	Moderate	Anomic
1022	M	70	17	42.5	35	74.2	59	Moderate	TMA
1001	F	52	17	13.125	84	73.8	59	Moderate	Conduction
0031	F	77	12	27.5	120	73.2	57	Moderate	Conduction
1023	F	61	19	27.5	104	71.9	55	Moderate	Wernicke's
1052	F	45	17	0	97	70.7	59	Moderate	Conduction
1044	M	57	19	10	87	70.3	58	Moderate	Conduction
1012	M	82	19	52.5	39	70	55	Moderate	Conduction
0025	F	49	12	18.75	20	69.9	58	Moderate	TMA
1031	F	60	19	28.75	94	69.9	57	Moderate	Broca's
1032	M	46	17	8.75	96	67.3	57	Moderate	Broca's
0009	M	63	14	27.5	176	64.9	50	Moderate	Broca's
1011	M	65	15	50	60	63.2	60	Moderate	Broca's
1036	M	67	17	42.5	49	62.8	52	Moderate	Broca's
1021	M	46	17	20	42	62	55	Moderate	Broca's
1046	M	70	13	27.5	43	61.8	53	Moderate	Wernicke's
0032	F	65	12	35	27	59.7	52	Moderate	TMA
1053	F	39	19	27.5	39	59.4	50	Moderate	Broca's
0018	F	49	16	6.89	31	55.9	50	Moderate	Broca's
1016	M	74	13	32.5	50	52.2	38	Moderate	Wernicke's
1017	M	77	21	45	103	38.6	50	Severe	Broca's
1027	M	66	17	26.25	35	37.7	25	Severe	Broca's
1034	M	59	15	32.5	203	36.8	34	Severe	Broca's
0029	M	59	16	26.89	13	36.1	34	Severe	Broca's
1048	M	72	17	30	13	28.9	36	Severe	Broca's
0022	F	58	18	39.38	129	23.6	36	V. Severe	Broca's
1024	M	66	16	28.75	73	17.9	26	V. Severe	Broca's
0028	F	64	15.5	23.13	7	16.3	23	V. Severe	Broca's

Abbreviations: Aud Comp, WAB‐R Auditory Word Recognition (single word comprehension; Ed., Education; F, Female; M, Male; max score = 60); Mo, months; PTA, Pure Tone Average; TPS, Time Post‐Stroke; Yr., years.

*PTA was calculated by averaging the hearing thresholds at 500 Hz, 1000 Hz, 2000 Hz, and 4000 Hz across both ears.

**1004 PTA was not collected due to an experimental error.

We additionally included 21 neurotypical control subjects (10 female) ranging in age from 24–77 years (*M =* 57.14, *SD =* 13.04). Control participants were native speakers of American English, had a mean of 16 (*SD =* 3.26) years of education, and reported no history of neurological or major psychiatric disease. All control participants reported normal or corrected to normal vision. Hearing was screened bilaterally using pure tone audiometry, with thresholds consistent with functional hearing for the task (*M =* 20.39 dB HL, *SD =* 12.02). There were no significant group differences in age (Welch's *t(*30) = −0.70, *p =* 0.50), years of education (Welch's *t(*30) = −0.90, *p =* 0.40), or hearing thresholds (Welch's *t(*33) = −2.00, *p =* 0.05) between the control and stroke participants.

The control group's cognitive status was assessed using the *Montreal Cognitive Assessment (MoCA*; Nasreddine et al. 2005). Seven participants scored below the standard cutoff of 24 (Julayanont et al. 2015); these participants additionally completed the *Cognitive Linguistic Quick Test‐Plus (CLQT+)*, with all scoring within normal limits, and were therefore retained in the sample. Control participants were also screened prior to enrollment for uncontrolled medical conditions with potential cognitive or neurological impact (e.g., hypertension, diabetes). Only participants with no such conditions or with conditions that were medically controlled were tested. Written informed consent was obtained from all participants. Purdue University and Midwestern University's Institutional Review Boards approved all study procedures.

### 2.2 Sentences

The sentence stimuli consisted of four declarative sentence structures, two canonical (C1, C2) and two non‐canonical (NC1, NC2; Wilson et al. 2010). Each sentence was comprised of 10 syllables, contained two nouns (boy, girl), one of seven verbs (kick, wash, chase, push, kiss, pull, hug), and one of three colors (blue, green, red). All sentences were spoken by a trained female vocalist using natural speech prosody (LaCroix et al. 2019, LaCroix et al. 2020). Table 2 contains a description and example of each syntax.

**TABLE 2:** Sentence stimuli examples and descriptions.

Sentence Structure	Example	Syntactic Parsing	Linguistic Features	Thematic Foil	Color Foil	Stimulus Duration
C1	The boy who is blue is pushing the girl.	The main clause follows subject–verb–object word order (The boy … is pushing the girl). The sentence contains a relative clause (who is blue) that follows canonical subject–verb word order and modifies the subject of the main clause (boy).	Subject‐modifying subject relative clause Right‐branching structure Embedded subject extraction Canonical thematic order	The image depicts the incorrect subject performing the action (girl) and the incorrect object receiving the action (boy), which conflicts with the interpretation of the main clause.	The image depicts incorrect color assignment to the object (girl), rather than the subject (boy), which conflicts with interpretation of the relative clause.	3.81–4.35 (*M* = 4.07, *sd* = 0.13)
C2	The boy is pushing the girl who is blue.	The main clause follows subject–verb–object word order (The boy is pushing the girl…). The sentence contains a relative clause (who is blue), which follows canonical subject‐verb word order and modifies the object of the main clause (girl).	Object‐modifying subject relative clause Right‐branching structure Embedded subject extraction Canonical thematic order	The image depicts the incorrect subject performing the action (girl) and the incorrect object receiving the action (boy), which conflicts with the interpretation of the main clause.	The image depicts incorrect color assignment to the subject (boy), rather than the object (girl), which conflicts with interpretation of the relative clause.	3.81–4.35 (*M =* 4.07, *sd* = 0.13)
NC1	The girl who the boy is pushing is blue.	The main clause follows subject–verb word order (The boy … is blue). The sentence contains an object relative clause (who the girl is pushing), where the object relative pronoun (who)—referring to *the girl* in this example—has been fronted to the beginning of the relative clause, resulting in an object–subject–verb word order within that clause.	Subject‐modifying object‐relative clause (active) Right‐branching structure Embedded object extraction Non‐canonical thematic order	The image depicts the incorrect subject performing the action (girl) and the incorrect object receiving the action (boy), which conflicts with the interpretation of the relative clause.	The image depicts incorrect color assignment by coloring the subject (boy) instead of the object (girl), which conflicts with the interpretation of the main clause.	3.88–4.73 (*M =* 4.29, *sd* = 0.15)
NC2	The boy who the girl is pushed by is blue.	The main clause follows subject–verb word order (The boy … is blue). The sentence contains a passive object relative clause (who the girl is pushed by), where the object (who) has been fronted to the beginning of the relative clause, resulting in an object–subject–verb word order. The relative clause modifies the subject of the main clause (boy).	Subject‐modifying object‐relative clause (passive) Right‐branching structure Embedded extraction from a by‐phrase Passive morphology in the relative clause Non‐canonical thematic order	The image depicts the incorrect subject performing the action (girl) and the incorrect object receiving the action (boy), which conflicts with the interpretation of the relative clause.	The image depicts incorrect color assignment by coloring the object (girl) instead of the subject (boy), which conflicts with the interpretation of the main clause.	3.88–4.73 (*M =* 4.29, *sd* = 0.15)

*Note*: All sentences were declarative and matched for syllable count (10 syllables per sentence). NC2 sentences contain one additional word relative to the other conditions due to the inclusion of passive morphology and preposition stranding (by). Thematic foils depict a reversal of the subject and object roles, but the color is assigned to the correct role. Color foils, on the other hand, assign the color to the incorrect subject or object while maintaining the subject/object roles consistent with the target sentence. Stimuli durations are reported in seconds.

### 2.3 Sentence‐Picture Matching Task

**FIGURE 1:**
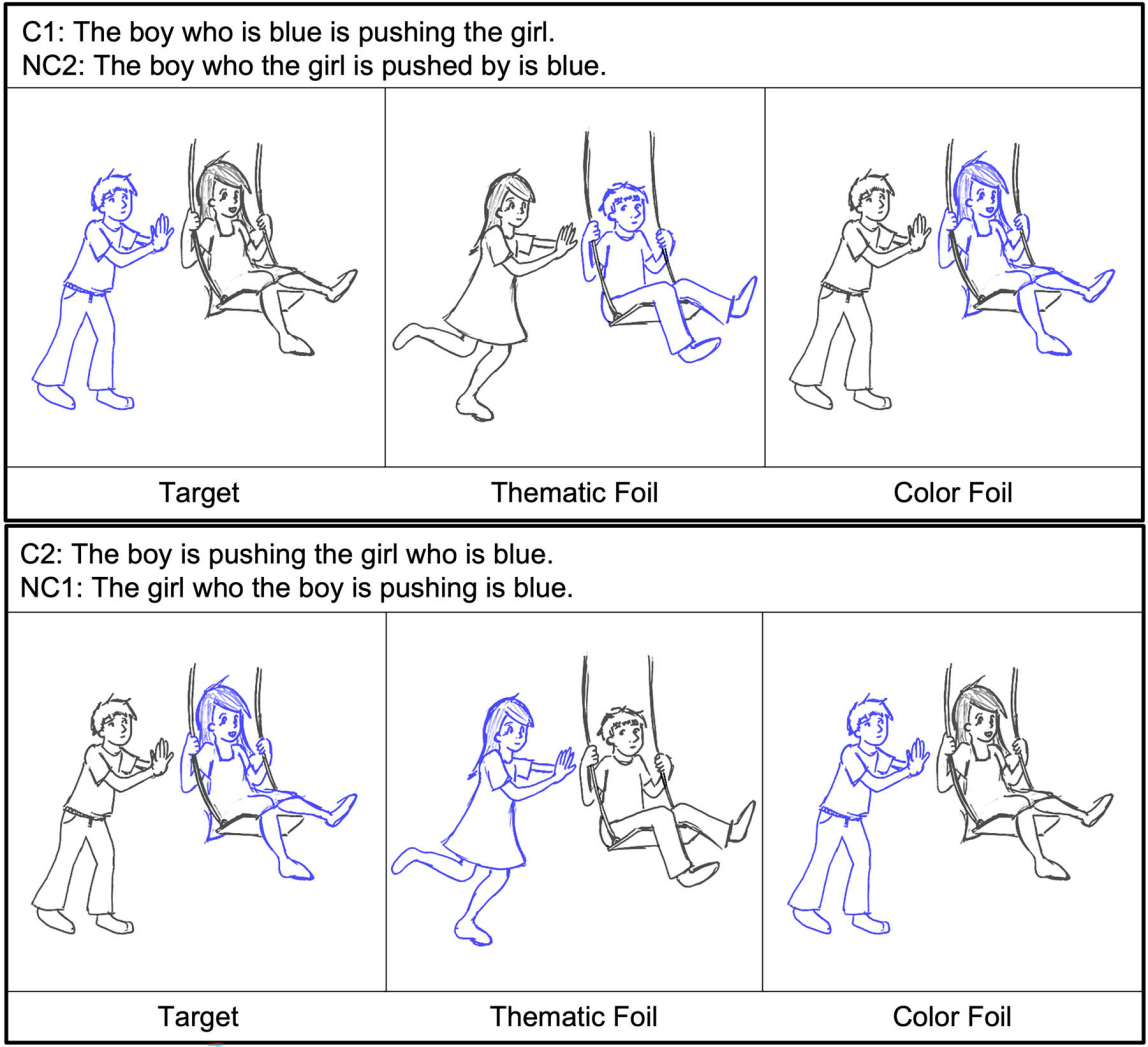
Examples of the visual stimuli for each sentence structure. The target image matches the auditorily presented sentence. Thematic foils depict a reversal of the subject and object roles but keep the correct color assignment for each role. In contrast, color foils maintain the correct subject‐object roles but assign colors incorrectly to one of the characters.

Sentence comprehension was measured using a sentence‐picture matching task (LaCroix et al. 2019, LaCroix et al. 2020; Wilson et al. 2010). Sixteen PWS and the control group completed the task using E‐Prime 3.0 (Psychology Research Tools, Inc, 2016). The other 48 PWS completed the task using Gorilla Experiment Builder (Anwyl‐Irvine et al. 2020). The timing and structure of each task were identical. Each trial began with the simultaneous presentation of an auditory sentence and two pictures. Sentences were presented binaurally at a volume audible but comfortable to each participant. One picture was the target and matched the auditory sentence. The other picture was a foil and either depicted a thematic role reversal or assigned the color to the wrong subject or object^1^ (Figure 1, Table 2). Participants were instructed to select the image that matched the auditory sentence as quickly and accurately as possible using a keyboard button press. Accuracy and reaction time were collected per trial. Stimulus presentation was randomized for all participants. Participants received written and verbal instructions and practice trials prior to the onset of the task.

While the timing and structure of each task was identical, the number of trials differed across the two platforms. The Gorilla task included 5 practice trials and 70 experimental trials (17 trials for the C1 and NC1 sentences, 18 trials for the C2 and NC2 sentences). The E‐Prime task contained 3 practice trials and 80 experimental trials, but only 40 of the experimental trials (10 per syntax) were spoken with a natural prosody; the other 40 trials were spoken with a list‐like prosody and were therefore excluded from the analyses. Accuracy (*t*(61) *=* 0.03, *p =* 0.98) and reaction time (*t*(61) *=* 1.67, *p =* 0.11) did not differ between the two platforms for PWS. This similarity justified combining the datasets, enabling a more comprehensive analysis of how aphasia severity and type affect sentence comprehension as syntactic complexity increases.

### 2.4 Statistical Analyses

All data were analyzed using RStudio 4.2.2 (R Core Team 2024). We first computed Pearson correlations to identify relationships between aphasia severity, measured using the *WAB‐R* aphasia quotient, and comprehension of each sentence structure in accuracy and reaction time. Next, we computed linear mixed‐effects models which allowed us to explore how sentence comprehension varied by aphasia severity and type within accuracy and reaction time.

All trials were included in the accuracy analyses. Trials associated with incorrect responses were excluded from the reaction time analyses; 13.69% of trials were excluded for the control group and 34.68% for PWS. The reaction time data were log‐transformed to better meet the assumption of normality. For accuracy, a mixed effects logistic regression was specified using the ‘glmer’ function from the *lme4* package (Bates et al. 2015) with a binomial distribution and a logit link. For reaction time, a linear mixed effects model was computed using the ‘lmer’ function from the *lme4* package (Bates et al. 2015).

The accuracy and reaction time models were each run within aphasia severity and type. For aphasia severity, the fixed effects were syntax (C1, C2, NC1, NC2) and aphasia severity (Control, Latent, Mild, Moderate, Severe), as well as their interaction. The aphasia type models were the same, except aphasia type (Control, Latent, Anomic, Conduction, Broca's) was substituted for aphasia severity. Age, education, and hearing acuity were examined as potential covariates; however, their inclusion did not improve model fit and they were therefore excluded from the final models (see Supplementary Materials, Section 2).

The random‐effects structure for all models was specified following the recommendations of Barr et al. (2013). Models were initially fit with a maximal random‐effects structure, including random intercepts and random slopes for all within‐subject effects, and were simplified only as necessary to achieve convergence and stable estimation. Full model specifications, including final random‐effects structures, are provided in Supplementary Tables 4–7. Briefly, the final reaction time models included a random intercept for subject, whereas the accuracy models included random intercepts for both subject and item.

Model results were first summarized using the ‘Anova’ function from the *car* package (Fox and Weisberg 2019). The *emmeans* package (Lenth 2025) was then used to explore custom contrasts of interest. Multiple comparisons were corrected using the Benjamini‐Hochberg (BH) false discovery rate (FDR; Benjamini and Hochberg 1995).

## 3 Results

### 3.1 Aphasia Severity

**FIGURE 2:**
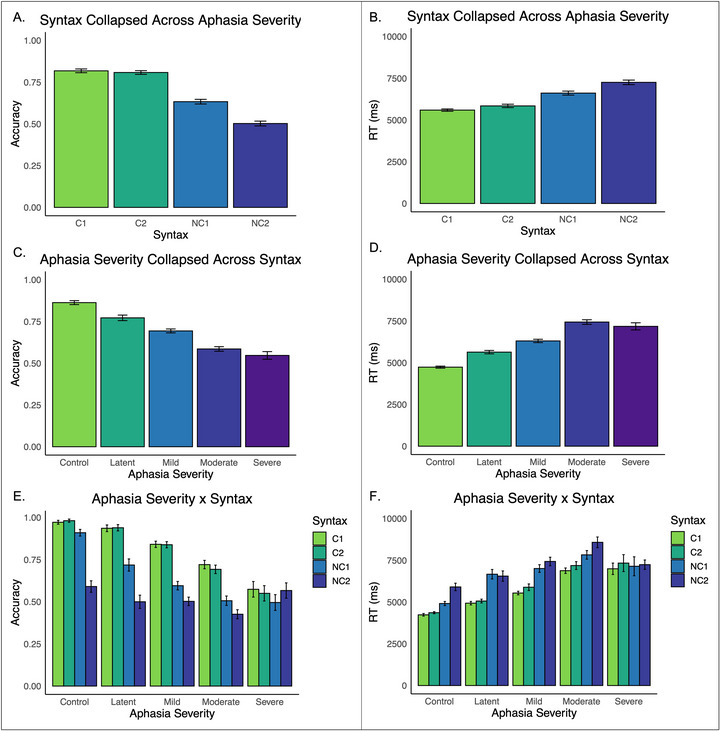
Means for each sentence structure in accuracy (A) and reaction time (B). Means for each aphasia severity within accuracy (C) and reaction time (D). Means for each sentence structure within each aphasia severity in accuracy (E) and reaction time (F). Means are presented in their original scales. Error bars represent ± one standard error.

All 64 PWS and 21 control participants were included in the aphasia severity analyses. Based on the *WAB‐R*, 10 PWS were characterized as having latent aphasia, 25 as mild aphasia, 21 as moderate aphasia, 5 as severe aphasia and 3 as very severe aphasia (Table 1). Because the severe and very severe aphasia groups each had small sample sizes, we combined them into a single group of 8 participants, referred to as the ‘severe aphasia’ group. Figure 2 and Supplementary Table 8 present descriptive statistics for each sentence structure, broken down by aphasia severity.

#### 3.1.1 Aphasia Severity Correlates With Sentence Comprehension

**FIGURE 3:**
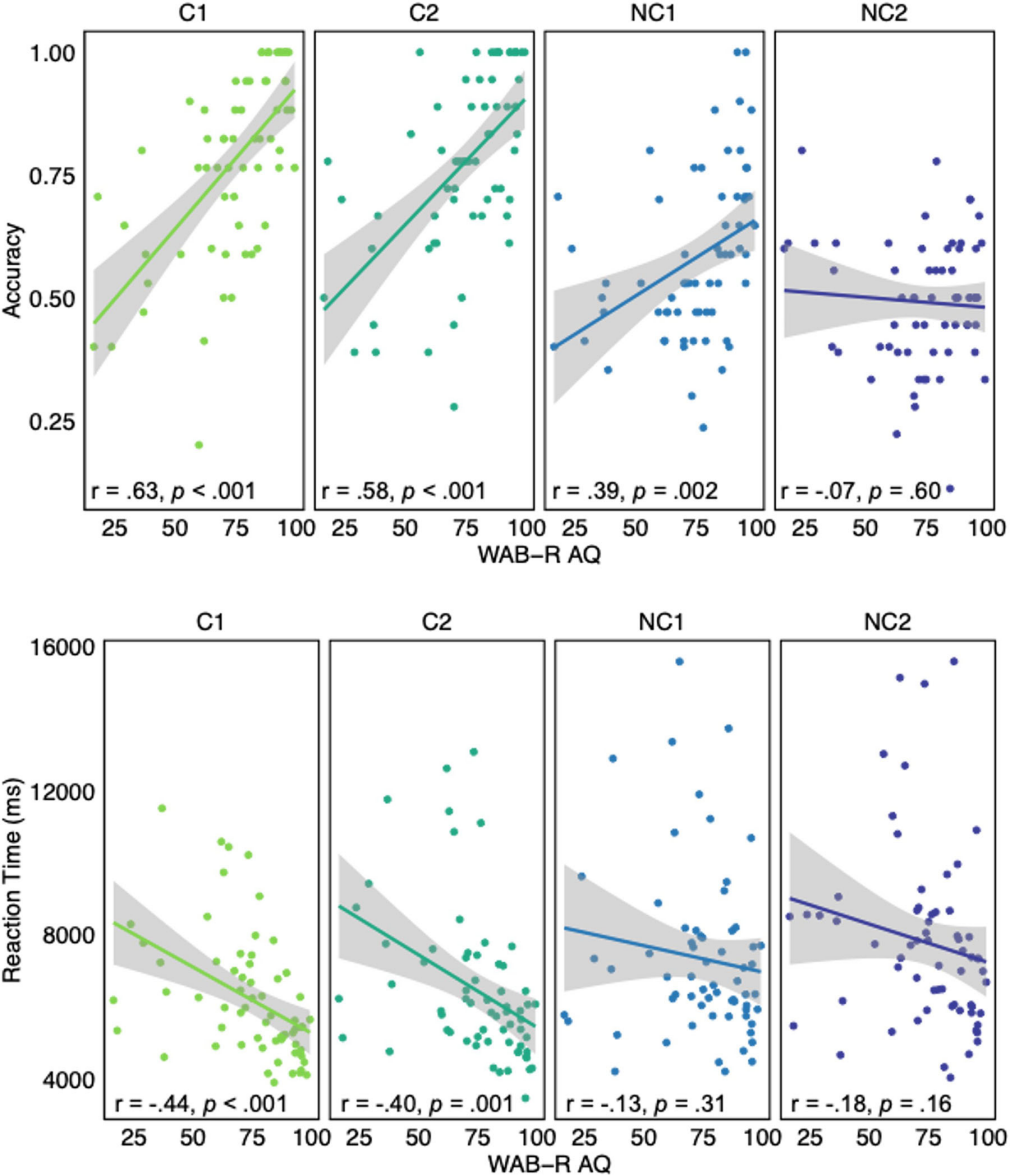
Plots depicting the correlations between aphasia severity (i.e., WAB‐R AQ) and each sentence structure in accuracy (top panel) and reaction time (bottom panel). Error bars represent ± one standard error.

Within PWS, there was a positive correlation between aphasia severity and sentence comprehension accuracy; PWS with higher *WAB‐R* aphasia quotients (i.e., more mild aphasia) demonstrated more accurate sentence comprehension for the C1, C2, and NC1 sentences, but not the NC2 sentences (Figure 3). We also observed a negative correlation between aphasia severity and reaction time: PWS with higher *WAB‐R* aphasia quotients demonstrated faster comprehension of the C1 and C2 sentences, but not the NC1 and NC2 sentences (Figure 3).

#### 3.1.2 Aphasia Severity Predicts Sentence Comprehension Accuracy

The fixed effect of syntax was significant, χ^2^(3) = 110.30, *p <* 0.001: all sentence structures differed from each other except the C1 and C2 sentences (Table 3; Figure 2A). The fixed effect of aphasia severity was also significant, χ^2^(4) = 79.80, *p <* 0.001: all groups differed from each other except the moderate and severe aphasia groups, which likely did not differ due to floor effects (Table 3; Figure 2C).

**TABLE 3:** Pairwise comparisons associated with the fixed effects of syntax and aphasia severity within accuracy and reaction time.

	**Accuracy**				**Reaction Time**			
	OR	*SE*	*z*	*p_FDR_*	β	*SE*	*t*	*p_FDR_*
	**Syntax**							
C1 vs. C2	0.96	0.18	−0.24	0.81	−0.02	0.02	−1.59	0.11
C1 vs. NC1	3.06	0.48	7.11	<0.001^*^	−0.12	0.02	−7.71	<0.001^*^
C1 vs. NC2	6.05	0.91	11.92	<0.001^*^	−0.20	0.02	−12.03	<0.001^*^
C2 vs. NC1	3.20	0.53	6.99	<0.001^*^	−0.10	0.02	−6.23	<0.001^*^
C2 vs. NC2	6.33	1.02	11.48	<0.001^*^	−0.17	0.02	−10.60	<0.001^*^
NC1 vs. NC2	1.98	0.24	5.60	<0.001^*^	−0.07	0.02	−4.35	<0.001^*^
	**Aphasia Severity**							
Control vs. Latent	2.56	0.67	3.61	<0.001^*^	−0.16	0.08	−2.03	0.06
Control vs. Mild	4.95	1.08	7.37	<0.001^*^	−0.24	0.06	−4.02	<0.001^*^
Control vs. Moderate	9.10	1.99	10.10	<0.001^*^	−0.45	0.06	−7.18	<0.001^*^
Control vs. Severe	10.90	2.72	9.58	<0.001^*^	−0.37	0.08	−4.39	<0.001^*^
Latent vs. Mild	0.52	0.10	3.41	0.001^*^	−0.08	0.08	−1.10	0.30
Latent vs. Moderate	0.28	0.06	6.49	<0.001^*^	−0.29	0.08	−3.77	0.001^*^
Latent vs. Severe	0.23	0.05	6.30	<0.001^*^	−0.21	0.10	−2.22	0.05^*^
Mild vs. Moderate	1.84	0.25	4.56	<0.001^*^	−0.21	0.06	−3.49	0.002^*^
Mild vs. Severe	2.20	0.40	4.37	<0.001^*^	−0.13	0.08	−1.57	0.15
Moderate vs. Severe	1.20	0.22	0.99	0.32	0.08	0.08	0.94	0.35

* Significant at *pFDR* ≤ 0.05.

The interaction between syntax and aphasia severity was also significant, χ^2^(12) = 118.20, *p <* 0.001. Here, we describe how sentence comprehension compared across adjacent groups within each syntax (i.e., control vs. latent, latent vs. mild, mild vs. moderate, moderate vs. severe; Figure 2E), however, Table 4 includes all possible aphasia severity comparisons and Supplementary Table 9 reports sentence comprehension differences within each group. Within the C1 and C2 sentences, there were no differences between the control and latent aphasia groups, but the latent aphasia group was more accurate than the mild aphasia group, the mild aphasia group was more accurate than the moderate aphasia group, and the moderate aphasia group was more accurate than the severe aphasia group. For the NC1 sentences, the control group was more accurate than the latent aphasia group, the latent aphasia group was more accurate than the mild aphasia group, the mild aphasia group was more accurate than the moderate aphasia group, but there were no differences in accuracy across the moderate and severe aphasia groups. No group differences were observed within the NC2 sentences.

**TABLE 4:** Pairwise comparisons for aphasia severity within each sentence structures for accuracy and reaction time.

	**Accuracy**				**Reaction Time**			
	OR	*SE*	*z*	*p_FDR_*	β	*SE*	*t*	*p_FDR_*
	**C1 Sentences**							
Control vs. Latent	2.45	1.35	1.64	0.10	−0.15	0.08	−1.83	0.09
Control vs. Mild	6.76	3.07	4.20	<0.001^*^	−0.25	0.06	−3.90	<0.001^*^
Control vs. Moderate	14.68	6.63	5.95	<0.001^*^	−0.50	0.07	−7.48	<0.001^*^
Control vs. Severe	28.22	13.60	6.93	<0.001^*^	−0.50	0.09	−5.45	<0.001^*^
Latent vs. Mild	2.75	1.04	2.67	0.01^*^	−0.10	0.08	−1.25	0.24
Latent vs. Moderate	5.99	2.24	4.78	<0.001^*^	−0.35	0.08	−4.28	<0.001^*^
Latent vs. Severe	11.51	4.76	5.91	<0.001^*^	−0.35	0.10	−3.40	0.002^*^
Mild vs. Moderate	2.17	0.47	3.61	0.001^*^	−.25	0.06	−3.96	<0.001^*^
Mild vs. Severe	4.18	1.16	5.16	<0.001^*^	−0.25	0.09	−2.81	0.008^*^
Moderate vs. Severe	1.92	0.52	2.43	0.02^*^	0.0005	0.09	0.006	1.00
	**C2 Sentences**							
Control vs. Latent	3.24	2.01	1.90	0.058	−0.12	0.08	−1.48	0.16
Control vs. Mild	9.62	5.16	4.22	<0.001^*^	−0.25	0.06	−3.92	<0.001^*^
Control vs. Moderate	23.71	12.60	5.94	<0.001^*^	−0.46	0.07	−6.93	<0.001^*^
Control vs. Severe	44.19	24.70	6.77	<0.001^*^	−0.48	0.09	−5.28	<0.001^*^
Latent vs. Mild	2.97	1.12	2.88	0.005^*^	−0.13	0.08	−1.63	0.13
Latent vs. Moderate	7.31	2.72	5.34	<0.001^*^	−0.34	0.08	−4.19	<0.001^*^
Latent vs. Severe	13.62	5.60	6.35	<0.001^*^	−0.36	0.10	−3.53	0.001^*^
Mild vs. Moderate	2.47	0.52	4.32	<0.001^*^	−0.21	0.06	−3.36	0.002^*^
Mild vs. Severe	4.59	1.25	5.61	<0.001^*^	0.23	0.09	−2.63	0.01^*^
Moderate vs. Severe	1.86	0.49	2.34	0.02^*^	−0.02	0.09	−0.23	0.82
	**NC1 Sentences**							
Control vs. Latent	3.86	1.31	3.98	<0.001^*^	−0.24	0.08	−2.92	0.01^*^
Control vs. Mild	6.77	1.98	6.52	<0.001^*^	−0.29	0.07	−4.50	<0.001^*^
Control vs. Moderate	10.16	3.02	7.79	<0.001^*^	−0.45	0.07	−6.66	<0.001^*^
Control vs. Severe	10.57	3.66	6.82	<0.001^*^	−0.27	0.09	−2.94	0.01^*^
Latent vs. Mild	1.75	0.43	2.28	0.03^*^	−0.05	0.08	−0.61	0.68
Latent vs. Moderate	2.64	0.66	3.85	<0.001^*^	−0.21	0.08	−2.53	0.02^*^
Latent vs. Severe	2.74	0.85	3.25	0.002^*^	−0.03	0.10	−0.28	0.83
Mild vs. Moderate	1.50	0.28	2.18	0.04^*^	−0.16	0.07	−2.47	0.02^*^
Mild vs. Severe	1.56	0.41	1.72	0.10	0.02	0.09	0.22	0.83
Moderate vs. Severe	1.04	0.28	0.15	0.88	0.18	0.09	1.95	0.08
	**NC2 Sentences**							
Control vs. Latent	1.39	0.37	1.26	0.34	−0.12	0.09	−1.38	0.24
Control vs. Mild	1.37	0.30	1.46	0.32	−0.18	0.07	−2.62	0.02^*^
Control vs. Moderate	1.94	0.43	2.96	0.03^*^	−0.39	0.07	−5.49	<0.001^*^
Control vs. Severe	1.07	0.30	0.25	0.90	−0.23	0.09	−2.48	0.03^*^
Latent vs. Mild	0.98	0.23	−0.08	0.94	−0.06	0.08	−0.69	0.54
Latent vs. Moderate	1.39	0.33	1.40	0.32	−0.27	0.09	−3.13	0.007^*^
Latent vs. Severe	0.77	0.23	−0.89	0.47	−0.11	0.11	−1.07	0.36
Mild vs. Moderate	1.41	0.26	1.90	0.19	−0.21	0.07	−3.18	0.007^*^
Mild vs. Severe	0.78	0.20	−0.96	0.47	−0.05	0.09	−0.61	0.54
Moderate vs. Severe	0.55	0.15	−2.26	0.12	0.16	0.09	1.70	0.15

* Significant at *pFDR* ≤ 0.05.

#### 3.1.3 Aphasia Severity Predicts Sentence Comprehension Reaction Time

The fixed effect of syntax was significant, F(3, 3241.4) = 31.81, *p <* 0.001: all sentence structures differed from each other except the C1 and C2 sentences (Table 3; Figure 2B). The fixed effect of aphasia severity was also significant, F(4, 102.7) = 17.02, *p <* 0.001. All groups differed from each other, except for the following comparisons: control versus latent aphasia, latent aphasia versus mild aphasia, mild aphasia versus severe aphasia, and moderate aphasia versus severe aphasia (Table 3; Figure 2D).

Syntax and aphasia severity interacted, F(12, 3241.5) = 4.77, *p <* 0.001. We report differences in adjacent groups within each syntax here (i.e., control vs. latent, latent vs. mild, mild vs. moderate, moderate vs. severe; Figure 2F), however, Table 4 includes all possible comparisons and Supplementary Table 9 reports sentence comprehension differences within each group. Within the C1, C2 and NC2 sentences, there were no differences between the control and latent aphasia groups, latent aphasia and mild aphasia groups, and moderate and severe aphasia groups, but the mild aphasia group was faster than the moderate aphasia group. For the NC1 sentences, the control group was faster than the latent aphasia group and the mild aphasia group was faster than the moderate aphasia group, but there were no differences between the latent and mild aphasia groups, nor between the moderate and severe aphasia groups.

### 3.2 Aphasia Type

**FIGURE 4:**
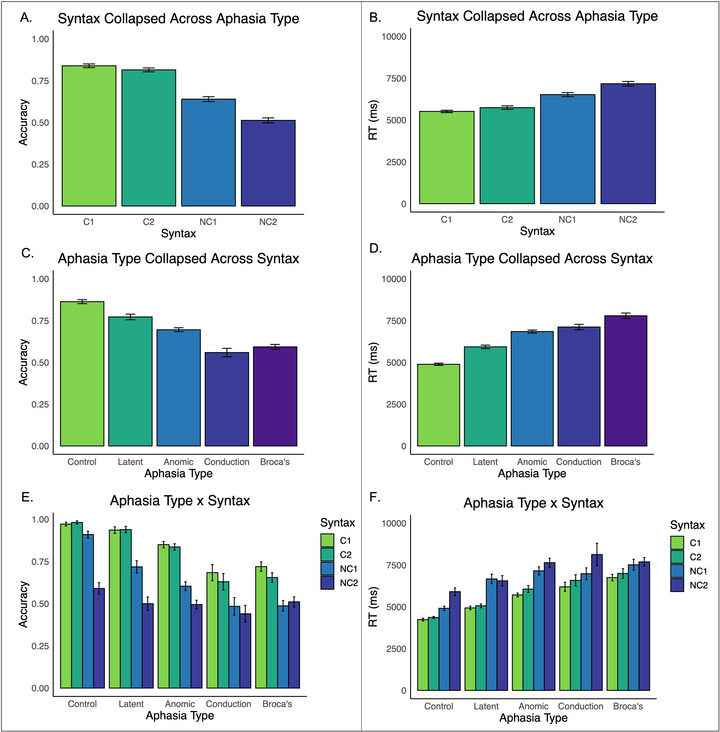
Means for each sentence structure in accuracy (A) and reaction time (B). Means for each aphasia type within accuracy (C) and reaction time (D). Means for each sentence structure within each aphasia type in accuracy (E) and reaction time (F). Means are presented in their original scales. Error bars represent ± one standard error.

The control group (*n* = 21) plus 58 PWS were included in this analysis. Based on the *WAB‐R*, 10 PWS had latent aphasia, 24 had anomic aphasia, 6 had conduction aphasia, and 18 had Broca's aphasia. Figure 4 and Supplementary Table 10 present descriptive statistics for each sentence structure, broken down by aphasia type.

**FIGURE 5:**
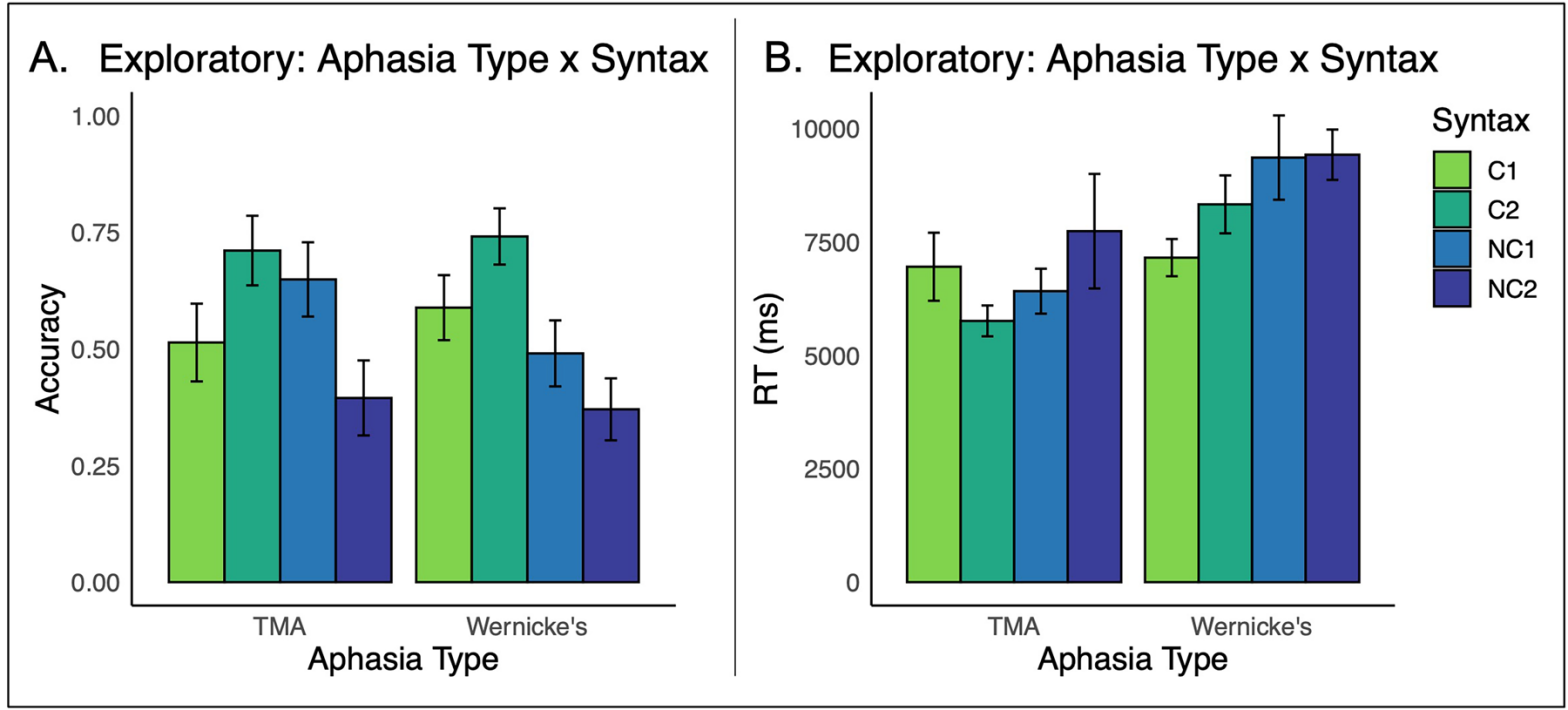
Sentence comprehension across the C1, C2, NC1, and NC2 sentences for participants with transcortical motor aphasia (TMA; *n* = 3) and Wernicke's aphasia (*n* = 5). Means are presented in their original scales. These groups were excluded from our main aphasia type analysis due to their small sample sizes.

PWS with Wernicke's (*n* = 3) and transcortical motor aphasia (*n* = 3) were excluded from these analyses due to their small sample sizes, but their data is graphed in Figure 5.

#### 3.2.1 Accuracy

The fixed effect of syntax was significant, χ^2^(3) = 110.60, *p <* 0.001: all sentence structures differed from each other except the C1 and C2 sentences (Table 5, Figure 4A). The fixed effect of aphasia type was also significant, χ^2^(4) = 59.50, *p <* 0.001: all aphasia types differed from each other except for the Broca's and conduction aphasia groups (Table 5, Figure 4D).

**TABLE 5:** Pairwise comparisons associated with the fixed effects of syntax and aphasia type within accuracy and reaction time.

	**Accuracy**				**Reaction Time**			
	OR	*SE*	*z*	*p_FDR_*	β	*SE*	*t*	*p_FDR_*
	**Syntax**							
C1 vs. C2	1.04	0.20	0.18	0.86	−0.03	0.02	−1.93	0.05^*^
C1 vs. NC1	3.48	0.56	7.76	<0.001^*^	−0.15	0.02	−9.11	<0.001^*^
C1 vs. NC2	7.04	1.09	12.64	<0.001^*^	−0.22	0.02	−12.64	<0.001^*^
C2 vs. NC1	3.36	0.57	7.17	<0.001^*^	−0.12	0.02	−7.29	<0.001^*^
C2 vs. NC2	6.80	1.11	11.75	<0.001^*^	−0.19	0.02	−10.89	<0.001^*^
NC1 vs. NC2	2.02	0.25	5.66	<0.001^*^	−0.07	0.02	−3.69	<0.001^*^
	**Aphasia Type**							
Control vs. Latent	2.55	0.67	3.56	<0.001^*^	−0.16	0.08	−1.97	0.08
Control vs. Anomic	4.88	1.08	7.20	<0.001^*^	−0.26	0.06	−4.24	<0.001^*^
Control vs. Conduction	10.41	2.79	8.73	<0.001^*^	−0.38	0.10	−3.88	<0.001^*^
Control vs. Broca's	8.78	1.97	9.68	<0.001^*^	−0.39	0.07	−5.82	<0.001^*^
Latent vs. Anomic	1.91	0.38	3.26	0.001^*^	−0.11	0.08	−1.37	0.22
Latent vs. Conduction	4.07	1.02	5.62	<0.001^*^	−0.22	0.11	−2.02	0.08
Latent vs. Broca's	3.44	0.70	6.08	<0.001^*^	−0.24	0.08	−2.85	0.01^*^
Anomic vs. Conduction	2.13	0.44	3.70	<0.001^*^	−0.11	0.10	−1.16	0.28
Anomic vs. Broca's	1.80	0.26	4.08	<0.001^*^	−0.13	0.07	−1.95	0.08
Conduction vs. Broca's	0.84	0.18	−0.82	0.41	−0.02	0.10	−0.16	0.87

* Significant at *pFDR* ≤ 0.05.

Syntax interacted with aphasia type, χ^2^(12) = 99.10, *p <* 0.001. Here, we focus on how aphasia type compared within each sentence structure, while Supplementary Table 11 presents differences in sentence structure performance within each aphasia type. In the C1 and C2 sentences, the control group was more accurate than all other groups except the latent aphasia group, which they did not differ from. The latent aphasia group outperformed the anomic, conduction, and Broca's aphasia groups. The anomic group was more accurate than the conduction and Broca's groups, while the conduction and Broca's groups did not differ. For the NC1 sentences, the control group outperformed all others. The latent aphasia group was more accurate than the anomic, conduction, and Broca's groups. The anomic group outperformed the Broca's group but did not differ from the conduction group. The conduction and Broca's groups did not differ. No significant group differences were observed within the NC2 sentences (Table 6; Figure 4E).

**TABLE 6:** Pairwise comparisons for aphasia type within sentence structures for accuracy and reaction time.

	**Accuracy**				**Reaction Time**			
	OR	*SE*	*z*	*p_FDR_*	β	*SE*	*t*	*p_FDR_*
	**C1 Sentences**							
Control vs. Latent	2.40	1.33	1.60	0.12	−0.15	0.08	−1.78	0.11
Control vs. Anomic	6.20	2.85	3.97	<0.001^*^	−0.28	0.07	−4.20	<0.001^*^
Control vs. Conduction	17.60	8.89	5.67	<0.001^*^	−0.40	0.10	−3.89	<0.001^*^
Control vs. Broca's	14.30	6.54	5.81	<0.001^*^	−0.47	0.07	−6.53	<0.001^*^
Latent vs. Anomic	2.60	0.99	2.46	0.02^*^	−0.13	0.08	−1.56	0.15
Latent vs. Conduction	7.30	3.19	4.55	<0.001^*^	−0.25	0.11	−2.20	0.05^*^
Latent vs. Broca's	5.90	2.26	4.66	<0.001^*^	−0.32	0.09	−3.68	0.001^*^
Anomic vs. Conduction	2.80	0.89	3.32	0.001^*^	−0.12	0.10	−1.21	0.26
Anomic vs. Broca's	2.30	0.53	3.62	<0.001^*^	−0.19	0.07	−2.76	0.01^*^
Conduction vs. Broca's	0.80	0.25	−0.67	0.51	−0.07	0.11	−0.65	0.52
	**C2 Sentences**							
Control vs. Latent	3.30	2.04	1.91	0.062	−0.12	0.08	−1.43	0.18
Control vs. Anomic	9.90	5.35	4.26	<0.001^*^	−0.28	0.07	−4.17	<0.001^*^
Control vs. Conduction	32.40	18.70	6.04	<0.001^*^	−0.42	0.10	−4.05	<0.001^*^
Control vs. Broca's	28.20	15.10	6.21	<0.001^*^	−0.43	0.07	−5.98	<0.001^*^
Latent vs. Anomic	3.00	1.15	2.91	0.005^*^	−0.16	0.08	−1.90	0.09
Latent vs. Conduction	9.90	4.25	5.32	<0.001^*^	−0.30	0.11	−2.61	0.02^*^
Latent vs. Broca's	8.60	3.25	5.68	<0.001^*^	−0.31	0.09	−3.57	0.002^*^
Anomic vs. Conduction	3.30	0.98	3.94	<0.001^*^	−0.14	0.10	−1.40	0.18
Anomic vs. Broca's	2.80	0.62	4.75	<0.001^*^	−0.15	0.07	−2.22	0.05^*^
Conduction vs. Broca's	0.90	0.26	−0.47	0.64	−0.01	0.11	−0.10	0.92
	**NC1 Sentences**							
Control vs. Latent	3.90	1.33	3.98	<0.001^*^	−0.24	0.09	−2.83	0.01^*^
Control vs. Anomic	6.60	1.95	6.36	<0.001^*^	−0.31	0.07	−4.66	<0.001^*^
Control vs. Conduction	11.30	4.17	6.57	<0.001^*^	−0.39	0.11	−3.65	0.001^*^
Control vs. Broca's	11.00	3.35	7.88	<0.001^*^	−0.36	0.07	−4.94	<0.001^*^
Latent vs. Anomic	1.70	0.42	2.09	0.046^*^	−0.07	0.08	−0.85	0.56
Latent vs. Conduction	2.90	0.97	3.21	0.002^*^	−0.14	0.12	−1.23	0.37
Latent vs. Broca's	2.88	0.74	3.97	<0.001^*^	−0.12	0.09	−1.35	0.36
Anomic vs. Conduction	1.70	0.49	1.89	0.07	−0.07	0.10	−0.70	0.56
Anomic vs. Broca's	1.70	0.33	2.57	0.01^*^	−0.05	0.07	−0.67	0.56
Conduction vs. Broca's	1.00	0.29	−0.09	0.93	0.02	0.11	0.23	0.82
	**NC2 Sentences**							
Control vs. Latent	1.40	0.37	1.22	0.55	−0.12	0.09	−1.34	0.26
Control vs. Anomic	1.40	0.31	1.54	0.55	−0.19	0.07	−2.79	0.02^*^
Control vs. Conduction	1.80	0.56	1.94	0.53	−0.30	0.11	−2.82	0.02^*^
Control vs. Broca's	1.30	0.31	1.26	0.55	−0.31	0.07	−4.17	<0.001^*^
Latent vs. Anomic	1.00	0.24	0.06	0.95	−0.08	0.09	−0.87	0.43
Latent vs. Conduction	1.30	0.42	0.87	0.55	−0.18	0.12	−1.55	0.21
Latent vs. Broca's	1.00	0.24	−0.13	0.95	−0.19	0.09	−2.14	0.09
Anomic vs. Conduction	1.30	0.37	0.93	0.55	−0.11	0.11	−1.03	0.38
Anomic vs. Broca's	1.00	0.19	−0.24	0.95	−0.12	0.07	−1.65	0.20
Conduction vs. Broca's	0.70	0.21	−1.06	0.55	−0.009	0.11	−0.09	0.93

* Significant at *pFDR* ≤ 0.05.

#### 3.2.2 Reaction Time

The fixed effect of syntax was significant, F(3, 3047.03) = 31.57, *p <* 0.001: all sentence structures differed from each other (Table 4; Figure 4B). The fixed effect of aphasia type was also significant, F(4, 91.99) = 12.16, *p <* 0.001: reaction times were comparable across all groups except the control group responded faster than the anomic, conduction, and Broca's aphasia groups, and the latent aphasia group responded faster than the Broca's aphasia group (Table 5; Figure 4D).

Syntax and aphasia type interacted, F(12, 3047.42) = 2.91, *p <* 0.001. Here, we report aphasia type comparisons within each sentence structure, while Supplementary Table 11 presents differences in sentence structure performance within each aphasia type. In the C1 and C2 sentences, the control group was faster than all other groups except the latent aphasia group. The latent aphasia group was faster than the conduction and Broca's aphasia groups but did not differ from the anomic group. The anomic group was faster than the Broca's group, while the conduction group did not differ from either the anomic or Broca's groups. In the NC1 sentences, all aphasia types were slower than the control group, but no differences were observed between the aphasia types. In the NC2 sentences, the control group was faster than the anomic, conduction, and Broca's groups but did not differ from the latent aphasia group. No other group differences were found. (Table 6, Figure 4F).

## 4 Discussion

Understanding how sentence comprehension is influenced by both aphasia type and severity is essential for developing more precise assessment and treatment strategies. This study extends prior work by jointly examining these factors in relation to syntactic complexity, revealing that left hemisphere stroke affects sentence comprehension, regardless of aphasia type or severity. Performance declined systematically with increasing syntactic complexity, underscoring that sentence‐level processing difficulties are pervasive, even among individuals not traditionally thought to exhibit comprehension deficits (e.g., latent and anomic aphasia).

Aphasia severity is a strong predictor of linguistic outcomes, with greater severity typically linked to more pronounced language impairments and poorer treatment responses (Fridriksson and Hillis 2021; Kristinsson et al. 2023; Nakagawa et al. 2019; Plowman et al. 2012). In the current study, sentence comprehension accuracy decreased as aphasia severity increased, consistent with prior findings (Klor and Mlchoh 1984; Murray 2018; Sung et al. 2024). Accuracy reductions were accompanied by slower reaction times for canonical sentences, though not for non‐canonical ones. Mixed‐effects model results confirmed that the mild aphasia group outperformed the moderate group in both accuracy and speed, while the moderate and severe groups differed only in accuracy for the canonical sentences.

The lack of differences between the moderate and severe aphasia groups on the non‐canonical sentences may reflect floor effects, as both groups performed near chance (∼50%) on the NC2 sentences. However, performance in the NC2 condition was generally low across severity levels, particularly in accuracy, suggesting that these sentences imposed substantial processing demands broadly rather than selectively overwhelming participants with more severe aphasia. Notably, while accuracy differences were minimal, reaction time differences persisted across severity levels, indicating continued divergence in processing efficiency even when accuracy approached floor levels. Non‐canonical constructions are known to increase working memory load, dependency integration demands, and attentional control requirements, even in neurotypical adults (e.g., Alexander 2006; Caplan and Waters 1999; January et al. 2009; Just and Carpenter 1992; Key‐DeLyria and Altmann 2016; King and Just 1991; Novick et al. 2005). Prior research further demonstrates that individuals with more severe aphasia show disproportionately poorer comprehension under heightened processing demands (Murray 2018), and computational models indicate that sentence comprehension deficits in aphasia may partly arise from reduced resource availability (Mätzig et al. 2018; Patil et al. 2016). Collectively, these findings suggest that performance in the moderate and severe aphasia groups is most consistent with an interaction between intrinsically high structural demands and diminished processing capacity. However, direct assessment of cognitive factors in future studies is needed to clarify this interpretation.

Building on prior work, the present findings also demonstrate that individuals with latent aphasia exhibit measurable sentence comprehension impairments relative to neurotypical controls, reinforcing evidence that subtle language deficits persist despite performance above clinical cutoffs. Such impairments have been documented across discourse, recall, and sentence processing (Boller 1968; Boller and Vignolo 1966; Bunker et al. 2025; Dalton and Richardson 2015; DeDe and Salis 2020; Fromm et al. 2017; Martzoukou et al. 2025; Newcombe and Marshall 1967; Salis et al. 2021; Salis and DeDe 2022; Zhang et al. 2024). Consistent with this literature, participants with latent aphasia showed reduced comprehension compared to controls across all but the most complex (NC2) sentences. These differences were most evident in accuracy, with comparatively limited divergence in reaction time, suggesting that comprehension deficits in latent aphasia reflect subtle reductions in successful interpretation rather than marked slowing of processing.

The latent aphasia group also differed from the anomic aphasia group, demonstrating higher accuracy across sentence types, though reaction times did not differ. Prior research has similarly found milder impairments in latent than anomic aphasia for tasks such as sentence recall (Salis et al. 2021) and discourse production (Dalton and Richardson 2015; Fromm et al. 2017). These findings add to the evidence suggesting that latent aphasia represents a distinct clinical profile rather than a milder form of anomic aphasia, and that standardized assessments such as the *WAB‐R* may lack the sensitivity to detect residual language deficits in this group.

The anomic aphasia group demonstrated lower accuracy than controls for all but the most complex (NC2) sentences and slower response times across all sentence types. Accuracy on the NC2 sentences approached chance across groups, consistent with floor effects driven by the high processing demands of this condition. Nevertheless, the anomic group remained slower than controls on these sentences, suggesting prolonged processing despite similarly low accuracy. In contrast, for the simpler sentence types, both accuracy and response time differences were evident, aligning with prior reports of comprehension difficulties in anomic aphasia (DeDe 2012, 2013; Dick et al. 2001; Salis et al. 2021; Shewan and Canter 1971), including deficits comparable to those observed in Broca's and conduction aphasia (Akinina et al. 2021; Berndt et al. 1997; Dick et al. 2001; Peach et al. 1988). While often characterized by word‐finding difficulties, our findings add to the growing evidence that anomic aphasia also involves subtle sentence‐level processing deficits, underscoring the need for more precise diagnostic tools.

### 4.1 Contributions to Theoretical Accounts of Sentence Comprehension Deficits

Although this study was not designed to adjudicate among theoretical accounts, performance on the NC2 sentences offers insight into competing explanations of sentence comprehension deficits. These sentences feature an atypical but grammatically legal structure—a main clause combined with a passive object‐relative clause—that makes them informative for contrasting representational deficit accounts (Grodzinsky 1995, 2000) with cognitive resource limitation accounts (e.g., Caplan et al. 2007; Caplan and Waters 2013). If comprehension difficulties primarily stem from degraded syntactic representations, then all groups, regardless of age or other individual differences, should perform similarly poorly on the NC2 sentences, provided they have comparable educational backgrounds. In contrast, if comprehension depends more on the availability of processing resources, then performance should vary systematically with factors that influence cognitive capacity, such as age, aphasia severity, or overall processing efficiency. Supporting this latter account, younger adults with education levels comparable to the older adult controls generally perform well above chance on the NC2 sentences (∼70%; e.g., LaCroix et al. 2019; LaCroix and Ratiu 2025), whereas the older adults in this study approached chance. Given evidence that syntactic knowledge remains relatively stable across the lifespan (Samu et al. 2017; Shafto and Tyler 2014), and age‐related declines in sentence comprehension are commonly attributed to reduced processing efficiency rather than grammatical loss (Bopp and Verhaeghen 2005; Caplan et al. 2011; Just and Carpenter 1992; van Boxtel and Lawyer 2021), this pattern suggests that the NC2 sentences taxed older adults’ cognitive resources beyond their available capacity.

This reasoning extends to aphasia: people with aphasia generally retain core syntactic representations (Linebarger et al. 1983; Wilson and Saygın 2004; Wulfeck 1988; Zhang and Hinzen 2022), and our aphasia and control groups were matched on age and education. Given these similarities, both groups should possess comparable knowledge of the NC2 grammatical structure. Consistent with this, inclusion of age and education as covariates did not account for significant variance in NC2 performance. The aphasia groups’ near‐chance performance therefore likely reflects a limitation in the cognitive resources available for sentence processing rather than a deficit in syntactic knowledge itself. Furthermore, the presence of agrammatic comprehension patterns even in mild or latent aphasia—where grammatical knowledge is expected to be largely preserved—provides additional support for the cognitive resource limitation account.

Our findings also bear on distinctions between lexical and syntactic mapping deficits (Caplan et al. 1985; Marshall 1995; Rochon et al. 2005). Although single word comprehension varied with overall severity, including it as a covariate did not account for differences in sentence comprehension performance across groups (see Supplementary Materials, Section 3). Thus, reduced comprehension of structurally complex constructions cannot be attributed solely to impaired lexical mapping and is more consistent with a syntactic mapping deficit, likely reflecting increased structural integration demands in the context of reduced processing resources.

## 5 Clinical Implications

These findings highlight the importance of assessing sentence comprehension across a range of syntactic structures, particularly in aphasia types such as anomic and latent aphasia, where individuals may appear unimpaired on standard tests but still have difficulty understanding complex syntax. Standardized assessments should therefore include non‐canonical sentence types (e.g., passives, object‐relative clauses) to detect subtle comprehension deficits that can affect everyday communication, such as following multi‐step directions or interpreting embedded clauses.

When deficits are identified, intervention approaches should explicitly target syntactic comprehension, with treatment tailored to the individual's language profile. For individuals with Broca's and conduction aphasia, therapy may need to include both simple and complex sentence structures, whereas those with anomic or latent aphasia may benefit from a greater focus on complex syntax. Tailoring intervention to an individual's syntactic profile may strengthen sentence‐level processing and support more effective communication in daily contexts.

## 6 Limitations and Future Directions

A few limitations should be considered when interpreting the current findings. First, subgroup sample sizes were relatively small, limiting the ability to examine fine‐grained interactions among aphasia type, severity, and syntactic complexity. Sample size constraints also led to overlap across the type‐ and severity‐based analyses (e.g., the anomic and mild aphasia groups largely comprised the same participants; see Table 1). We report both sets of analyses to provide a comprehensive picture and to begin to integrate findings across previously separate lines of research. Larger and more diverse samples will enable higher‐resolution analyses of severity beyond *WAB‐R* categories, as substantial variability likely exists within the mild, moderate, and severe groupings. Relatedly, although combining data from two related studies improved statistical power, participants completed slightly different numbers of trials. Task and stimuli were otherwise identical, and no performance differences were observed across studies; nonetheless, replication using a single standardized protocol is recommended.

Finally, this study focused exclusively on sentence comprehension and did not include independent measures of cognition. Future work should incorporate assessments of attention and working memory to clarify their contributions to comprehension across aphasia types and severity levels. Additionally, aligning task‐based measures with self‐reported communication challenges could help determine the real‐world impact of subtle sentence comprehension deficits, particularly in latent and mild aphasia.

## 7 Conclusion

This study demonstrates that left hemisphere stroke can affect sentence comprehension even in individuals without a formal aphasia diagnosis and in aphasia types not typically associated with comprehension deficits. Differences between latent and anomic aphasia suggest that latent aphasia reflects a distinct clinical presentation rather than a milder variant. Across all types and severities, comprehension declined with increasing syntactic complexity. These findings underscore the importance of considering both aphasia type and severity in clinical evaluation and intervention and highlight the need for assessment tools and therapies that more effectively address sentence‐level processing across the aphasia spectrum.

## Funding

This work was supported by a 2023 ASHFoundation New Investigators Research Grant (PI: A. LaCroix) and the National Institutes of Health (R21DC021481; PI: A. LaCroix).

## Ethics Statement

All study procedures conformed to the Declaration of Helsinki and were approved by the Institutional Review Boards at Purdue University (2023‐1067) and Midwestern University (AZ 1464).

## Consent

Written informed consent was obtained from all participants prior to study participation.

## Conflicts of Interest

The authors declare no conflicts of interest.

## Supporting information




**Supplementary Table 1**. R code and model comparison results for mixed‐effects models with and without foil type added as a fixed effect (covariate).
**Supplementary Table 2**. R code and model comparison results for mixed‐effects models with and without covariates for age, education, and hearing thresholds.
**Supplementary Table 3**. R code and model comparison results for mixed‐effects models with and without single word comprehension covariate.
**Supplementary Table 4**. Full linear mixed‐effects model predicting accuracy as a function of syntax and aphasia severity, specified as: glmer(accuracy ∼ syntax * aphasia_severity + (1 | subject) + (1 | item), family = binomial(link = “logit”), control = glmerControl(optimizer = “bodyqa”)).
**Supplementary Table 5**. Full linear mixed‐effects model predicting log‐transformed reaction time as a function of syntax and aphasia severity, specified as: lmer(RT_log ∼ syntax * aphasia_severity + (1 | subject)).
**Supplementary Table 6**. Full linear mixed‐effects model predicting accuracy as a function of syntax and aphasia type, specified as: glmer(accuracy ∼ syntax * aphasia_type + (1 | subject) + (1 | item), family = binomial(link = “logit”), control = glmerControl(optimizer = “bodyqa”)).
**Supplementary Table 7**. Full linear mixed‐effects model predicting log‐transformed reaction time as a function of syntax and aphasia type, specified as: lmer(RT_log ∼ syntax * aphasia_type + (1 | subject)).
**Supplementary Table 8**. Means and standard errors for accuracy and reaction time are reported in each variable's original scale as *M (SE)*. Estimated marginal means from the linear mixed‐effects models are reported as *EMM (SE)*, along with their associated standard errors. Reaction times are log transformed in the linear mixed‐effects models. Groups are divided by aphasia severity.
**Supplementary Table 9**. Pairwise comparisons between sentence structures within each aphasia severity.
**Supplementary Table 10**. Means and standard errors for accuracy and reaction time are reported in each variable's original scale as *M (SE)*. Estimated marginal means from the linear mixed‐effects models are reported as *EMM (SE)*, along with their associated standard errors. Reaction times are log transformed in the linear mixed‐effects models. Groups are divided by aphasia type.
**Supplementary Table 11**. Pairwise comparisons between sentence structures within each aphasia type.

## Data Availability

The data supporting the findings are available from the corresponding author (Arianna N. LaCroix) upon reasonable request.

## Endnotes

1 The balance of foil types differed slightly between the two datasets. In the Purdue University dataset (n = 48), half of the foils were thematic, and half were color across all four syntactic conditions. In the Midwestern University dataset (n = 16), the C1 sentences had thematic foils, the C2 sentences had color foils, and for the NC1 and NC2 sentences, half of the foils were thematic, and half were color. Given these differences, we evaluated whether foil type influenced sentence processing in preliminary models (see Supplementary Materials, Section 1). Although inclusion of foil type improved reaction time model fit, it did not affect accuracy or alter the pattern of syntax or group effects in the reaction time models. Foil type was therefore excluded from the final models.
