# Supplementary material for: The Influence of Aphasia Type and Severity on Sentence Comprehension after Left Hemisphere Stroke
Source: Int J Lang Commun Disord. 2026 Apr 9;61:e70245. doi: 10.1111/1460-6984.70245 (PMC13064428; doi:10.1111/1460-6984.70245)
Supplement: Supplementary file 1 — Supplementary Table 1. R code and model comparison results for mixed‐effects models with and without foil type added as a fixed effect (covariate). Supplementary Table 2. R code and model comparison results for mixed‐effects models with and without covariates for age, education, and hearing thresholds. Supplementary Table 3. R code and model comparison results for mixed‐effects models with and without single word comprehension covariate. Supplementary Table 4. Full linear mixed‐effects model predicting accuracy as a function of syntax and aphasia severity, specified as: glmer(accuracy ∼ syntax * aphasia_severity + (1 | subject) + (1 | item), family = binomial(link = “logit”), control = glmerControl(optimizer = “bodyqa”)). Supplementary Table 5. Full linear mixed‐effects model predicting log‐transformed reaction time as a function of syntax and aphasia severity, specified as: lmer(RT_log ∼ syntax * aphasia_severity + (1 | subject)). Supplementary Table 6. Full linear mixed‐effects model predicting accuracy as a function of syntax and aphasia type, specified as: glmer(accuracy ∼ syntax * aphasia_type + (1 | subject) + (1 | item), family = binomial(link = “logit”), control = glmerControl(optimizer = “bodyqa”)). Supplementary Table 7. Full linear mixed‐effects model predicting log‐transformed reaction time as a function of syntax and aphasia type, specified as: lmer(RT_log ∼ syntax * aphasia_type + (1 | subject)). Supplementary Table 8. Means and standard errors for accuracy and reaction time are reported in each variable's original scale as M (SE). Estimated marginal means from the linear mixed‐effects models are reported as EMM (SE), along with their associated standard errors. Reaction times are log transformed in the linear mixed‐effects models. Groups are divided by aphasia severity. Supplementary Table 9. Pairwise comparisons between sentence structures within each aphasia severity. Supplementary Table 10. Means and standard errors for accuracy and reaction time a [file JLCD-61-0-s001.docx]

**Supplemental Materials:** Section 1 presents R code and model comparisons for foil analyses; Section 2 for covariate analyses; and Section 3 for single word comprehension analyses. Supplementary Tables 4-7 provide the complete mixed-effects model results, including fixed effects, random-effects structures, and model specifications. Supplementary Tables 8 and 10 provide means in the original scale, estimated marginal means, and their associated standard errors for the aphasia severity and type analyses, respectively. Supplementary Tables 9 and 11 report pairwise comparisons between each sentence structure within each aphasia severity and type, respectively. These results complement the aphasia severity x syntax and aphasia type x syntax interactions reported in the main text.

**Section 1: Evaluation of Foil Type**

Because the two datasets differed in the foils used for canonical sentences (see Footnote 1 in the main text), foil type (color vs. thematic) was examined as a potential covariate to determine whether it influenced syntactic processing or accounted for additional variance in reaction time or accuracy beyond the primary predictors of syntax and aphasia severity/type.

As an initial check, we conducted Welch two-sample *t*-tests comparing log-transformed reaction time and accuracy across foil types. These analyses did not reveal significant differences between color and thematic foils (log RT: *t*(167) = −1.0, *p* = .20; accuracy: *t*(160) = 2.0, *p* = .06).

To further evaluate the potential influence of foil type, we next incorporated it into the mixed-effects modeling framework. Given our primary theoretical interest in whether syntactic processing differs as a function of aphasia severity/type, foil type was first entered as part of a three-way interaction with syntax and aphasia severity/type. These models were rank-deficient, indicating insufficient information to estimate all interaction parameters reliably.

We then evaluated foil type as a fixed effect covariate. As shown in Supplementary Table 1, inclusion of foil type did not improve model fit for either accuracy model, but it did significantly improve fit for both reaction time models, reflecting faster responses to color than thematic foils. Importantly, inclusion of foil type did not alter the pattern or interpretation of the primary effects. Foil type was therefore excluded from the final reaction time models to maintain consistency across dependent variables.

| **Supplementary Table 1**. R code and model comparison results for mixed-effects models with and without foil type added as a fixed effect (covariate). | | | | | | |
| --- | --- | --- | --- | --- | --- | --- |
| **Aphasia Severity: Accuracy** | | | | | | |
| Model 1: base_model <- glmer(accuracy ~ syntax * aphasia_severity + (1 \| subject) + (1 \| item), family = binomial(link = “logit”), control = glmerControl(optimizer = “bodyqa”))  Model 2: foil_model <- glmer(accuracy ~ syntax * aphasia_severity + foil + (1 \| subject) + (1 \| item), family = binomial(link = “logit”), control = glmerControl(optimizer = “bodyqa”))  anova(base_model, foil_model) | | | | | | |
|  | npar | AIC | BIC | logLink | Deviance | χ^2^ |
| Model 1 | 22 | 5191 | 5334 | -2574 | 5147 | χ^2^(1) = 1.48, *p =* .22 |
| Model 2 | 23 | 5192 | 5341 | -2573 | 5166 |  |
| **Aphasia Severity: Log RT** | | | | | | |
| Model 1: base_model <- lmer(RT_log ~ syntax * aphasia_severity + (1 \| subject))  Model 2: foil_model <- lmer(RT_log ~ syntax * aphasia_severity + foil + (1 \| subject))  anova(base_model, foil_model) | | | | | | |
|  | npar | AIC | BIC | logLink | Deviance | χ^2^ |
| Model 1 | 22 | 1413 | 1547 | -684 | 1369 | χ^2^(1) = 12.20, *p <* .001* |
| Model 2 | 23 | 1402 | 1543 | -678 | 1356 |  |
| **Aphasia Type: Accuracy** | | | | | | |
| Model 1: base_model <- lmer(RT_log ~ syntax * aphasia_type + (1 \| subject))  Model 2: foil_model <- glmer(accuracy ~ syntax * aphasia_type + foil + (1 \| subject) + (1 \| item), family = binomial(link = “logit”), control = glmerControl(optimizer = “bodyqa”))  anova(base_model, foil_model) | | | | | | |
|  | npar | AIC | BIC | logLink | Deviance | χ^2^ |
| Model 1 | 22 | 4727 | 4868 | -2342 | 4683 | χ^2^(1) = 1.51, *p =* .22 |
| Model 2 | 23 | 4728 | 4875 | -2341 | 4682 |  |
| **Aphasia Type: Log RT** | | | | | | |
| Model 1: base_model <- lmer(RT_log ~ syntax * aphasia_type + (1 \| subject))  Model 2: foil_model <- lmer(RT_log ~ syntax * aphasia_type+ foil + (1 \| subject))  anova(base_model, foil_model) | | | | | | |
|  | npar | AIC | BIC | logLink | Deviance | χ^2^ |
| Model 1 | 22 | 1355 | 1488 | -656 | 1311 | χ^2^(1) = 6.96, *p =* .008* |
| Model 2 | 23 | 1350 | 1489 | -652 | 1304 |  |

**Section 2: Evaluation of Potential Covariates**

In preliminary analyses, we examined whether participant-level factors influenced performance. Age and education were included because they may index differences in cognitive reserve and processing efficiency, and hearing thresholds (pure-tone average; PTA) were included because variability in hearing sensitivity could affect performance on the auditory sentence comprehension task. Mixed-effects models were estimated with age, education, and PTA entered as fixed-effect covariates. Age and education were mean-centered, and PTA was standardized (z-scored) prior to analysis to facilitate model convergence and improve interpretability of parameter estimates. Covariates were added after establishing the random-effects structure described in the main text. As shown in Supplementary Table 2, inclusion of these variables did not significantly improve model fit and these variables were not retained in the final models.

| **Supplementary Table 2**. R code and model comparison results for mixed-effects models with and without covariates for age, education, and hearing thresholds. | | | | | | |
| --- | --- | --- | --- | --- | --- | --- |
| **Aphasia Severity: Accuracy** | | | | | | |
| Model 1: base_model <- glmer(accuracy ~ syntax * aphasia_severity + (1 \| subject) + (1 \| item), family = binomial(link = “logit”), control = glmerControl(optimizer = “bodyqa”))  Model 2: covariate_model <- glmer(accuracy ~ syntax * aphasia_severity + PTA_z + Age_c + Education_c + (1 \| subject) + (1 \| item), family = binomial(link = “logit”), control = glmerControl(optimizer = “bodyqa”))  anova(base_model, covariate_model) | | | | | | |
|  | npar | AIC | BIC | logLink | Deviance | χ^2^ |
| Model 1 | 22 | 5114 | 5256 | -2535 | 5070 | χ^2^(3) = 3.08, *p =* .38 |
| Model 2 | 25 | 5117 | 5279 | -2533 | 5067 |  |
| **Aphasia Severity: Log RT** | | | | | | |
| Model 1: base_model <- lmer(RT_log ~ syntax * aphasia_severity + (1 \| subject))  Model 2: covariate_model <- lmer(RT_log ~ syntax * aphasia_severity + PTA_z + Age_c + Education_c + (1 \| subject))  anova(base_model, covariate_model) | | | | | | |
|  | npar | AIC | BIC | logLink | Deviance | χ^2^ |
| Model 1 | 22 | 1416 | 1550 | -686 | 1372 | χ^2^(3) = 3.92, *p =* .27 |
| Model 2 | 25 | 1418 | 1570 | -684 | 1368 |  |
| **Aphasia Type: Accuracy** | | | | | | |
| Model 1: base_model <- lmer(RT_log ~ syntax * aphasia_type + (1 \| subject))  Model 2: covariate_model <- glmer(accuracy ~ syntax * aphasia_type + PTA_z + Age_c + Education_c + (1 \| subject) + (1 \| item), family = binomial(link = “logit”), control = glmerControl(optimizer = “bodyqa”))  anova(base_model, covariate_model) | | | | | | |
|  | npar | AIC | BIC | logLink | Deviance | χ^2^ |
| Model 1 | 22 | 4650 | 4790 | -2303 | 4606 | χ^2^(3) = 3.57, *p =* .31 |
| Model 2 | 25 | 4652 | 4812 | -2301 | 4602 |  |
| **Aphasia Type: Log RT** | | | | | | |
| Model 1: base_model <- lmer(RT_log ~ syntax * aphasia_type + (1 \| subject))  Model 2: foil_model <- lmer(RT_log ~ syntax * aphasia_type + PTA_z + Age_c + Education_c + (1 \| subject))  anova(base_model, covariate_model) | | | | | | |
|  | npar | AIC | BIC | logLink | Deviance | χ^2^ |
| Model 1 | 22 | 1358 | 1491 | -657 | 1314 | χ^2^(3) = 3.49, *p =* .32 |
| Model 2 | 25 | 1360 | 1511 | -655 | 1310 |  |
| *Note.* Hearing threshold data (PTA) were unavailable for one participant (1004). To permit valid likelihood-based model comparisons, models with and without covariates were fit on the subset of participants with complete data (n = 63). | | | | | | |

**Section 3: Role of Single Word Comprehension**

Accurate interpretation of sentence comprehension deficits requires distinguishing whether breakdown occurs at the level of individual word representations or at the level of syntactic structure. As noted by Caplan et al. (1985), single word comprehension is critical for differentiating lexical from syntactic sources of impairment during sentence processing. According to the mapping hypothesis (Marshall, 1995; Rochon et al., 2005), a lexical mapping deficit is characterized by impaired word-level comprehension, such that difficulty understanding individual words compromises accurate assignment of thematic roles within a sentence. In contrast, a syntactic mapping deficit is observed when word-level comprehension is relatively preserved, but performance declines for structurally complex sentences requiring computation and integration of non-canonical dependencies.

To determine whether sentence-level performance in the present study was driven by single word comprehension deficits, we examined *WAB-R Auditory Word Recognition* scores as a participant-level covariate in the mixed-effects models. This allowed us to assess whether single word comprehension accounted for unique variance in sentence accuracy or reaction time beyond the primary predictors of syntax and aphasia severity/type. The accuracy models (type and severity) did not converge when this measure was included. Both reaction time models converged (Supplementary Table 3); however, inclusion of single word comprehension did not significantly improve model fit. Accordingly, this variable was not retained in the final models.

| **Supplementary Table 3**. R code and model comparison results for mixed-effects models with and without single word comprehension covariate. | | | | | | |
| --- | --- | --- | --- | --- | --- | --- |
| **Aphasia Severity: Log RT** | | | | | | |
| Model 1: base_model <- lmer(RT_log ~ syntax * aphasia_severity + (1 \| subject))  Model 2: covariate_model <- lmer(RT_log ~ syntax * aphasia_severity + single word comp + (1 \| subject))  anova(base_model, covariate_model) | | | | | | |
|  | npar | AIC | BIC | logLink | Deviance | χ^2^ |
| Model 1 | 18 | 1362 | 1443 | -651 | 1303 | χ^2^(1) = 0.00, *p =* .99 |
| Model 2 | 19 | 1341 | 1451 | -651 | 1303 |  |
| **Aphasia Type: Log RT** | | | | | | |
| Model 1: base_model <- lmer(RT_log ~ syntax * aphasia_type + (1 \| subject))  Model 2: foil_model <- lmer(RT_log ~ syntax * aphasia_type+ single word comp + (1 \| subject))  anova(base_model, covariate_model) | | | | | | |
|  | npar | AIC | BIC | logLink | Deviance | χ^2^ |
| Model 1 | 18 | 1362 | 1466 | -633 | 1326 | χ^2^(3) = 3.49, *p =* .32 |
| Model 2 | 19 | 1364 | 1474 | -633 | 1326 |  |

| **Supplementary Table 4.** Full linear mixed-effects model predicting accuracy as a function of syntax and aphasia severity, specified as: glmer(accuracy ~ syntax * aphasia_severity + (1 \| subject) + (1 \| item), family = binomial(link = “logit”), control = glmerControl(optimizer = “bodyqa”)). | | | | |
| --- | --- | --- | --- | --- |
| ***Predictors*** | ***Estimate*** | ***SE*** | ***z*** | ***p*** |
| (Intercept) | 3.64 | 0.42 | **8.58** | **<0.001*** |
| Syntax [C2] | 0.36 | 0.66 | 0.55 | 0.585 |
| Syntax [NC1] | -1.30 | 0.49 | **-2.66** | **0.008*** |
| Syntax [NC2] | -3.29 | 0.44 | **-7.35** | **<0.001*** |
| Severity [Mild] | -1.91 | 0.45 | **-4.22** | **<0.001*** |
| Severity [Moderate] | -2.69 | 0.45 | **-5.98** | **<0.001*** |
| Severity [Severe] | -3.34 | 0.48 | **-6.96** | **<0.001*** |
| Severity [Latent] | -0.90 | 0.55 | -1.64 | 0.101 |
| Syntax [C2] × Severity [Mild] | -0.35 | 0.68 | -0.52 | 0.606 |
| Syntax [NC1] × Severity [Mild] | -0.002 | 0.52 | 0.00 | 0.998 |
| Syntax [NC2] × Severity [Mild] | 1.60 | 0.48 | 3.32 | **0.001*** |
| Syntax [C2] × Severity [Moderate] | -0.48 | 0.68 | -0.71 | 0.480 |
| Syntax [NC1] × Severity [Moderate] | 0.37 | 0.52 | 0.71 | 0.476 |
| Syntax [NC2] × Severity [Moderate] | 2.02 | 0.48 | **4.23** | **<0.001*** |
| Syntax [C2] × Severity [Severe] | -0.45 | 0.71 | -0.63 | 0.526 |
| Syntax [NC1] × Severity [Severe] | 0.98 | 0.55 | **1.77** | 0.077 |
| Syntax [NC2] × Severity [Severe] | 3.27 | 0.52 | **6.29** | **<0.001*** |
| Syntax [C2] × Severity [Latent] | -0.28 | 0.80 | -0.35 | 0.728 |
| Syntax [NC1] × Severity [Latent] | -0.45 | .61 | **-.74** | 0.462 |
| Syntax [NC2] × Severity [Latent] | 0.56 | .58 | 0.98 | 0.327 |
| **Random Effects** | | | | |
| Subject | 0.12 (0.34) | | | |
| Item | 0.06 (0.25) | | | |
| N _Subject_: 85 | N _Item_: 110 N _Observations_: 4840 | | | |
| *Note.* Estimates are from a binomial generalized linear mixed-effects model with a logit link, fit using maximum likelihood (Laplace approximation), with random intercepts for subject and item. Treatment coding was used (reference levels: C1 syntax; control severity).  * Significant at *p <* .05 | | | | |

| **Supplementary Table 5.** Full linear mixed-effects model predicting log-transformed reaction time as a function of syntax and aphasia severity, specified as: lmer(RT_log ~ syntax * aphasia_severity + (1 \| subject)). | | | | |
| --- | --- | --- | --- | --- |
| ***Predictors*** | ***Estimate*** | ***SE*** | ***t*** | ***p*** |
| (Intercept) | 8.32 | 0.05 | **175.01** | **<0.001*** |
| Syntax [C2] | 0.04 | 0.03 | 1.42 | 0.155 |
| Syntax [NC1] | 0.15 | 0.03 | **5.20** | **<0.001*** |
| Syntax [NC2] | 0.30 | 0.03 | **8.96** | **<0.001*** |
| Severity [Mild] | 0.25 | 0.06 | **3.90** | **<0.001*** |
| Severity [Moderate] | 0.50 | 0.07 | **7.48** | **<0.001*** |
| Severity [Severe] | 0.50 | 0.09 | **5.45** | **<0.001*** |
| Severity [Latent] | 0.15 | 0.08 | 1.83 | 0.067 |
| Syntax [C2] × Severity [Mild] | 0.00 | 0.04 | 0.02 | 0.984 |
| Syntax [NC1] × Severity [Mild] | 0.04 | 0.04 | 1.13 | 0.257 |
| Syntax [NC2] × Severity [Mild] | -0.07 | 0.04 | -1.74 | 0.081 |
| Syntax [C2] × Severity [Moderate] | -0.04 | 0.04 | -0.96 | 0.335 |
| Syntax [NC1] × Severity [Moderate] | -0.05 | 0.04 | -1.12 | 0.262 |
| Syntax [NC2] × Severity [Moderate] | -0.11 | 0.04 | **-2.52** | **0.012*** |
| Syntax [C2] × Severity [Severe] | -0.02 | 0.06 | -0.28 | 0.780 |
| Syntax [NC1] × Severity [Severe] | -0.23 | 0.06 | **-3.28** | **<0.001*** |
| Syntax [NC2] × Severity [Severe] | -0.27 | 0.06 | **-4.51** | **<0.001*** |
| Syntax [C2] × Severity [Latent] | -0.03 | 0.04 | -0.67 | 0.502 |
| Syntax [NC1] × Severity [Latent] | 0.09 | .05 | **2.01** | **0.045*** |
| Syntax [NC2] × Severity [Latent] | -0.03 | .05 | -0.61 | 0.544 |
| **Random Effects** | | | | |
| Residual | 0.08 (0.29) | | | |
| Subject | 0.04 (0.20) | | | |
| N _Subject_: 85 | N _Observations_: 3338 | | | |
| *Note.* Estimates are from a REML-fitted linear mixed-effects model with a subject-level random intercept. Treatment coding was used (reference levels: C1 syntax; control severity).  * Significant at *p <* .05 | | | | |

| **Supplementary Table 6.** Full linear mixed-effects model predicting accuracy as a function of syntax and aphasia type, specified as: glmer(accuracy ~ syntax * aphasia_type + (1 \| subject) + (1 \| item), family = binomial(link = “logit”), control = glmerControl(optimizer = “bodyqa”)). | | | | |
| --- | --- | --- | --- | --- |
| ***Predictors*** | ***Estimate*** | ***SE*** | ***z*** | ***p*** |
| (Intercept) | 3.64 | 0.43 | **8.48** | **<0.001*** |
| Syntax [C2] | 0.38 | 0.66 | 0.57 | 0.566 |
| Syntax [NC1] | -1.28 | 0.49 | **-2.61** | **0.009*** |
| Syntax [NC2] | -3.29 | 0.45 | **-7.28** | **<0.001*** |
| Type [Latent] | -0.88 | 0.55 | **-1.60** | 0.110 |
| Type [Anomic] | -1.83 | 0.46 | **-3.97** | **<0.001*** |
| Type [Conduction] | -2.87 | 0.51 | **-5.67** | **<0.001*** |
| Type [Broca's] | -2.66 | 0.46 | -5.81 | **<0.001*** |
| Syntax [C2] × Type [Latent] | -0.31 | 0.81 | -0.38 | 0.703 |
| Syntax [NC1] × Type [Latent] | -0.48 | 0.62 | -0.77 | 0.439 |
| Syntax [NC2] × Type [Latent] | 0.56 | 0.58 | 0.96 | 0.339 |
| Syntax [C2] × Type [Anomic] | -0.47 | 0.69 | -0.68 | 0.497 |
| Syntax [NC1] × Type [Anomic] | -0.06 | 0.53 | -0.11 | 0.910 |
| Syntax [NC2] × Type [Anomic] | 1.49 | 0.49 | **3.05** | **0.002*** |
| Syntax [C2] × Type [Conduction] | -0.61 | 0.73 | -0.84 | 0.402 |
| Syntax [NC1] × Type [Conduction] | 0.44 | 0.58 | **0.76** | 0.446 |
| Syntax [NC2] × Type [Conduction] | 2.27 | 0.55 | **4.16** | **<0.001*** |
| Syntax [C2] × Type [Broca's] | -0.68 | 0.69 | -0.99 | 0.323 |
| Syntax [NC1] × Type [Broca's] | 0.26 | 0.52 | **0.50** | 0.618 |
| Syntax [NC2] × Type [Broca's] | 2.37 | 0.49 | 4.86 | **<0.001*** |
| **Random Effects** | | | | |
| Subject | 0.13 (0.35) | | | |
| Item | 0.06 (0.25) | | | |
| N _Subject_: 79 | N _Item_: 110 N _Observations_: 4480 | | | |
| *Note.* Estimates are from a binomial generalized linear mixed-effects model with a logit link, fit using maximum likelihood (Laplace approximation), with random intercepts for subject and item. Treatment coding was used (reference levels: C1 syntax; control severity).  * Significant at *p <* .05 | | | | |

| **Supplementary Table 7.** Full linear mixed-effects model predicting log-transformed reaction time as a function of syntax and aphasia type, specified as: lmer(RT_log ~ syntax * aphasia_type + (1 \| subject)). | | | | |
| --- | --- | --- | --- | --- |
| ***Predictors*** | ***Estimate*** | ***SE*** | ***t*** | ***p*** |
| (Intercept) | 8.32 | 0.05 | **169.90** | **<0.001*** |
| Syntax [C2] | 0.04 | 0.03 | 1.42 | 0.156 |
| Syntax [NC1] | 0.15 | 0.03 | **5.18** | **<0.001*** |
| Syntax [NC2] | 0.30 | 0.03 | **8.93** | **<0.001*** |
| Type [Latent] | 0.15 | 0.08 | **1.78** | 0.079 |
| Type [Anomic] | 0.28 | 0.07 | **4.20** | **<0.001*** |
| Type [Conduction] | 0.40 | 0.10 | **3.88** | **<0.001*** |
| Type [Broca's] | 0.47 | 0.07 | 6.53 | **<0.001*** |
| Syntax [C2] × Type [Latent] | -0.03 | 0.04 | -0.67 | 0.503 |
| Syntax [NC1] × Type [Latent] | 0.09 | 0.05 | 2.00 | **0.046*** |
| Syntax [NC2] × Type [Latent] | -0.03 | 0.05 | -0.60 | 0.546 |
| Syntax [C2] × Type [Anomic] | -0.00 | 0.04 | -0.05 | 0.957 |
| Syntax [NC1] × Type [Anomic] | 0.04 | 0.04 | 0.93 | 0.354 |
| Syntax [NC2] × Type [Anomic] | -0.08 | 0.04 | **-2.00** | **0.046*** |
| Syntax [C2] × Type [Conduction] | 0.02 | 0.06 | 0.31 | 0.759 |
| Syntax [NC1] × Type [Conduction] | -0.01 | 0.06 | **-0.23** | 0.822 |
| Syntax [NC2] × Type [Conduction] | -0.10 | 0.07 | **-1.51** | 0.131 |
| Syntax [C2] × Type [Broca's] | -0.04 | 0.04 | -0.95 | 0.341 |
| Syntax [NC1] × Type [Broca's] | -0.11 | 0.04 | **-2.44** | **0.015*** |
| Syntax [NC2] × Type [Broca's] | -0.16 | 0.05 | -3.43 | **0.001*** |
| **Random Effects** | | | | |
| Residual | 0.08 (0.29) | | | |
| Subject | 0.04 (0.21) | | | |
| N _Subject_: 79 | N _Observations_: 3138 | | | |
| *Note.* Estimates are from a REML-fitted linear mixed-effects model with a subject-level random intercept. Treatment coding was used (reference levels: C1 syntax; control severity).  * Significant at *p <* .05 | | | | |

| **Supplementary Table 8.** Means and standard errors for accuracy and reaction time are reported in each variable’s original scale as *M (SE)*. Estimated marginal means from the linear mixed-effects models are reported as *EMM (SE)*, along with their associated standard errors. Reaction times are log transformed in the linear mixed-effects models. Groups are divided by aphasia severity. | | | | | |
| --- | --- | --- | --- | --- | --- |
|  | Accuracy | | Reaction Time | |  |
|  | *M (SE)* | *EMM (SE)* | *M (SE)* | *EMM (SE)* |  |
| **Control Group** | | | | | |
| C1 | .97 (.01) | 3.65 (.43) | 4234.91 (78.71) | 8.32 (.05) |  |
| C2 | .98 (.01) | 4.00 (.51) | 4362.27 (62.09) | 8.36 (.05) |  |
| NC1 | .91 (.02) | 2.35 (.26) | 4911.18 (123.28) | 8.47 (.05) |  |
| NC2 | .59 (.03) | .36 (.18) | 5905.04 (223.67) | 8.62 (.05) |  |
| **Latent Aphasia** | | | | | |
| C1 | .94 (.02) | 2.75 (.35) | 4932.20 (104.47) | 8.47 (.07) |  |
| C2 | .94 (.02) | 2.83 (.35) | 5059.57 (113.52) | 8.49 (.07) |  |
| NC1 | .72 (.04) | 1.00 (.22) | 6666.23 (284.50) | 8.72 (.07) |  |
| NC2 | .50 (.04) | .02 (.20) | 6553.44 (309.27) | 8.74 (.07) |  |
| **Mild Aphasia** | | | | | |
| C1 | .84 (.02) | 1.74 (.17) | 5538.61 (107.83) | 8.57 (.04) |  |
| C2 | .84 (.02) | 1.74 (.16) | 5886.28 (197.87) | 8.61 (.04) |  |
| NC1 | .60 (.03) | .44 (.14) | 7006.94 (230.80) | 8.77 (.04) |  |
| NC2 | .50 (.02) | .04 (.13) | 7430.65 (253.98) | 8.80 (.04) |  |
| **Moderate Aphasia** | | | | | |
| C1 | .72 (.03) | .96 (.16) | 6876.32 (164.19) | 8.82 (.05) |  |
| C2 | .69 (.03) | .84 (.15) | 7185.47 (234.45) | 8.83 (.05) |  |
| NC1 | .51 (.03) | .03 (.15) | 7825.64 (258.27) | 8.93 (.05) |  |
| NC2 | .43 (.03) | -.31 (.14) | 8576.15 (320.04) | 9.01 (.05) |  |
| **Severe Aphasia** | | | | | |
| C1 | .57 (.05) | .31 (.23) | 6991.38 (341.89) | 8.82 (.08) |  |
| C2 | .55 (.05) | .22 (.23) | 7331.72 (505.92) | 8.85 (.08) |  |
| NC1 | .50 (.05) | -.01 (.23) | 7141.87 (567.95) | 8.75 (.08) |  |
| NC2 | .57 (.05) | .29 (.23) | 7239.31 (281.20) | 8.85 (.08) |  |

| **Supplementary Table 9.** Pairwise comparisons between sentence structures within each aphasia severity. | | | | | | | | |
| --- | --- | --- | --- | --- | --- | --- | --- | --- |
|  | **Accuracy** | | | | **Reaction Time** | | | |
|  | OR | *SE* | *z* | *p_FDR_* | β | *SE* | *t* | *p_FDR_* |
|  | **Control Group** | | | | | | | |
| C1 vs. C2 | .70 | .46 | -.54 | .59 | -.04 | .03 | -1.42 | .22 |
| C1 vs. NC1 | 3.66 | 1.79 | 2.65 | .01* | -.15 | .03 | -5.20 | <.001* |
| C1 vs. NC2 | 26.86 | 12.10 | 7.31 | <.001* | -.30 | .03 | -9.00 | <.001* |
| C2 vs. NC1 | 5.23 | 2.97 | 2.92 | .005* | -.11 | .03 | -3.81 | <.001* |
| C2 vs. NC2 | 38.42 | 20.50 | 6.84 | <.001* | -.26 | .03 | -7.75 | <.001* |
| NC1 vs. NC2 | 7.35 | 2.20 | 6.65 | <.001* | -.15 | .03 | -4.34 | <.001* |
|  | **Latent Aphasia** | | | | | | | |
| C1 vs. C2 | .92 | .43 | -.17 | .87 | -.01 | .03 | -.33 | .80 |
| C1 vs. NC1 | 5.75 | 2.19 | 4.59 | <.001* | -.24 | .04 | -6.74 | <.001* |
| C1 vs. NC2 | 15.27 | 5.67 | 7.34 | <.001* | -.26 | .04 | -6.65 | <.001* |
| C2 vs. NC1 | 6.22 | 2.37 | 4.80 | <.001* | -.23 | .04 | -6.50 | <.001* |
| C2 vs. NC2 | 16.52 | 6.13 | 7.56 | <.001* | -.25 | .04 | -6.43 | <.001* |
| NC1 vs. NC2 | 2.66 | .67 | 3.87 | <.001* | -.02 | .04 | -.49 | .69 |
|  | **Mild Aphasia** | | | | | | | |
| C1 vs. C2 | 1.00 | .21 | -.02 | .98 | -.04 | .02 | -1.84 | .11 |
| C1 vs. NC1 | 3.66 | .70 | 6.77 | <.001* | -.19 | .03 | -7.76 | <.001* |
| C1 vs. NC2 | 5.45 | 1.03 | 8.96 | <.001* | -.22 | .03 | -8.58 | <.001* |
| C2 vs. NC1 | 3.68 | .70 | 6.91 | <.001* | -.10 | .03 | -3.44 | .002* |
| C2 vs. NC2 | 5.47 | 1.02 | 9.14 | <.001* | -.18 | .03 | -5.83 | <.001* |
| NC1 vs. NC2 | 1.49 | .25 | 2.41 | .02* | -.03 | .03 | -1.02 | .39 |
|  | **Moderate Aphasia** | | | | | | | |
| C1 vs. C2 | 1.13 | .21 | .64 | .52 | -.003 | .03 | -.10 | .95 |
| C1 vs. NC1 | 2.53 | .47 | 5.03 | <.001* | -.10 | .03 | -3.53 | .001* |
| C1 vs. NC2 | 3.55 | .65 | 6.90 | <.001* | -.18 | .03 | -5.93 | <.001* |
| C2 vs. NC1 | 2.24 | .40 | 4.48 | <.001* | -.10 | .03 | -3.44 | .002* |
| C2 vs. NC2 | 3.14 | .56 | 6.40 | <.001* | -.18 | .03 | -5.83 | <.001* |
| NC1 vs. NC2 | 1.40 | .25 | 1.93 | .065 | -.08 | .03 | -2.35 | .03* |
|  | **Severe Aphasia** | | | | | | | |
| C1 vs. C2 | 1.09 | .30 | .33 | .94 | -.02 | .05 | -.48 | .70 |
| C1 vs. NC1 | 1.37 | .38 | 1.13 | .83 | .08 | .05 | 1.48 | .20 |
| C1 vs. NC2 | 1.02 | .28 | .07 | .94 | -.03 | .05 | -.51 | .68 |
| C2 vs. NC1 | 1.25 | .34 | .82 | .83 | .10 | .05 | 1.95 | .08 |
| C2 vs. NC2 | .93 | .26 | -.26 | .94 | -.001 | .05 | -.03 | .99 |
| NC1 vs. NC2 | .74 | .21 | -1.07 | .83 | -.10 | .05 | -1.99 | .08 |
| * Significant at *p_FDR_ <* .05. | | | | | | | | |

| **Supplementary Table 10.** Means and standard errors for accuracy and reaction time are reported in each variable’s original scale as *M (SE)*. Estimated marginal means from the linear mixed-effects models are reported as *EMM (SE)*, along with their associated standard errors. Reaction times are log transformed in the linear mixed-effects models. Groups are divided by aphasia type. | | | | |
| --- | --- | --- | --- | --- |
|  | Accuracy | | Reaction Time | |
|  | *M (SE)* | *EMM (SE)* | *M (SE)* | *EMM (SE)* |
| **Control Group** | | | | |
| C1 | .97 (.01) | 3.64 (.43) | 4234.91 (78.71) | 8.32 (.05) |
| C2 | .98 (.01) | 4.02 (.52) | 4362.27 (62.09) | 8.36 (.05) |
| NC1 | .91 (.02) | 2.36 (.27) | 4911.18 (123.28) | 8.47 (.05) |
| NC2 | .59 (.03) | .35 (.18) | 5905.04 (223.67) | 8.62 (.05) |
| **Latent Aphasia** | | | | |
| C1 | .94 (.02) | 2.76 (.35) | 4932.20 (104.47) | 8.47 (.07) |
| C2 | .94 (.02) | 2.83 (.35) | 4362.27 (62.09) | 8.49 (.07) |
| NC1 | .72 (.04) | 1.00 (.22) | 6666.23 (284.50) | 8.72 (.07) |
| NC2 | .50 (.04) | .03 (.20) | 6553.44 (309.27) | 8.74 (.07) |
| **Anomic Aphasia** | | | | |
| C1 | .85 (.02) | 1.81 (.17) | 5709.07 (113.03) | 8.60 (.05) |
| C2 | .84 (.02) | 1.72 (.17) | 6059.80 (208.35) | 8.64 (.05) |
| NC1 | .60 (.03) | .47 (.14) | 7150.22 (237.30) | 8.79 (.05) |
| NC2 | .50 (.03) | .01 (.14) | 7640.46 (264.98) | 8.81 (.05) |
| **Conduction Aphasia** | | | | |
| C1 | .68 (.05) | .77 (.27) | 6195.87 (271.15) | 8.73 (.09) |
| C2 | .63 (.05) | .54 (.26) | 6579.17 (335.89) | 8.78 (.09) |
| NC1 | .48 (.05) | -.07 (.26) | 6978.19 (350.61) | 8.86 (.09) |
| NC2 | .44 (.05) | -.25 (.26) | 8127.76 (670.06) | 8.92 (.09) |
| **Broca’s Aphasia** | | | | |
| C1 | .72 (.03) | .98 (.17) | 6745.87 (191.69) | 8.79 (.05) |
| C2 | .65 (.03) | .68 (.16) | 6994.74 (292.29) | 8.79 (.05) |
| NC1 | .49 (.03) | -.04 (.16) | 7510.82 (325.28) | 8.84 (.05) |
| NC2 | .51 (.03) | .06 (.16) | 7687.14 (253.21) | 8.93 (.05) |

| Supplementary Table 11. Pairwise comparisons between sentence structures within each aphasia type. | | | | | | | | |
| --- | --- | --- | --- | --- | --- | --- | --- | --- |
|  | **Accuracy** | | | | **Reaction Time** | | | |
|  | OR | *SE* | *z* | *p_FDR_* | β | *SE* | *t* | *p_FDR_* |
|  | **Control Group** | | | | | | | |
| C1 vs. C2 | .68 | .46 | -.57 | .57 | -.04 | .03 | -1.42 | .24 |
| C1 vs. NC1 | 3.59 | 1.76 | 2.61 | .01* | -.15 | .03 | -5.18 | <.001* |
| C1 vs. NC2 | 26.74 | 12.10 | 7.28 | <.001* | -.30 | .03 | -8.93 | <.001* |
| C2 vs. NC1 | 5.25 | 2.98 | 2.91 | .005* | -.11 | .03 | -3.80 | <.001* |
| C2 vs. NC2 | 39.05 | 20.90 | 6.86 | <.001* | -.26 | .03 | -7.72 | <.001* |
| NC1 vs. NC2 | 7.44 | 2.23 | 6.69 | <.001* | -.14 | .03 | -4.32 | <.001* |
|  | **Latent Aphasia** | | | | | | | |
| C1 vs. C2 | .93 | .44 | -.15 | .88 | -.01 | .03 | -.33 | .81 |
| C1 vs. NC1 | 5.80 | 2.21 | 4.62 | <.001* | -.24 | .04 | -6.71 | <.001* |
| C1 vs. NC2 | 15.35 | 5.70 | 7.36 | <.001* | -.26 | .04 | -6.63 | <.001* |
| C2 vs. NC1 | 6.23 | 2.37 | 4.80 | <.001* | -.23 | .04 | -6.48 | <.001* |
| C2 vs. NC2 | 16.48 | 6.11 | 7.55 | <.001* | -.25 | .04 | -6.41 | <.001* |
| NC1 vs. NC2 | 2.65 | .67 | 3.86 | <.001* | -.02 | .04 | -.49 | .70 |
|  | **Anomic Aphasia** | | | | | | | |
| C1 vs. C2 | 1.10 | .24 | .42 | .67 | -.04 | .02 | -1.67 | .16 |
| C1 vs. NC1 | 3.81 | .75 | 6.77 | <.001* | -.19 | .03 | -7.31 | <.001* |
| C1 vs. NC2 | 6.05 | 1.18 | 9.24 | <.001* | -.21 | .03 | -7.90 | <.001* |
| C2 vs. NC1 | 3.48 | .67 | 6.52 | <.001* | -.15 | .03 | -5.84 | <.001* |
| C2 vs. NC2 | 5.53 | 1.04 | 9.07 | <.001* | -.17 | .03 | -6.50 | <.001* |
| NC1 vs. NC2 | 1.59 | .27 | 2.76 | .007* | -.02 | .03 | -.84 | .50 |
|  | **Conduction Aphasia** | | | | | | | |
| C1 vs. C2 | 1.26 | .40 | .74 | .54 | -.06 | .05 | -1.14 | .35 |
| C1 vs. NC1 | 2.31 | .73 | 2.66 | .02* | -.14 | .06 | -2.45 | .03* |
| C1 vs. NC2 | 2.77 | .86 | 3.28 | .006* | -.20 | .06 | -3.46 | .002* |
| C2 vs. NC1 | 1.83 | .56 | 1.99 | .07 | -.08 | .06 | -1.39 | .25 |
| C2 vs. NC2 | 2.20 | .66 | 2.61 | .02* | -.01 | .06 | -2.40 | .03* |
| NC1 vs. NC2 | 1.20 | .36 | .61 | .54 | -.06 | .06 | -.98 | .43 |
|  | **Broca’s Aphasia** | | | | | | | |
| C1 vs. C2 | 1.35 | .27 | 1.50 | .16 | -.001 | .03 | -.04 | .97 |
| C1 vs. NC1 | 2.77 | .55 | 5.13 | <.001* | -.04 | .03 | -1.34 | .27 |
| C1 vs. NC2 | 2.51 | .49 | 4.68 | <.001* | -.14 | .03 | -4.31 | <.001* |
| C2 vs. NC1 | 2.05 | .39 | 3.75 | <.001* | -.04 | .03 | -1.29 | .29 |
| C2 vs. NC2 | 1.86 | .35 | 3.28 | .002* | -.14 | .03 | -4.22 | <.001* |
| NC1 vs. NC2 | .91 | .17 | -.53 | .60 | -.09 | .03 | -2.69 | .02* |
| * Significant at *p_FDR_ <* .05. | | | | | | | | |

**References**

Caplan, D., Baker, C., & Dehaut F. (1985). Syntactic determinants of sentence comprehension in aphasia. Cognition, 21,117-175.

Marshall, J. (1995). The mapping hypothesis and aphasia therapy. Aphasiology, 9(6), 517–539.

Rochon, E., Laird, L., Bose, A. & Scofield, J. (2005). Mapping therapy for sentence production impairments in nonfluent aphasia. Neuropsychological Rehabilitation, 15, 1-36.
